# A randomized crossover study to evaluate local tolerability following subcutaneous administration of a new depot medroxyprogesterone acetate contraceptive formulation^☆^

**DOI:** 10.1016/j.conx.2023.100100

**Published:** 2023-09-12

**Authors:** Vera Halpern, Angie Wheeless, Vivian Brache, Anja Lendvay, Leila Cochón, Douglas Taylor, Laneta J. Dorflinger

**Affiliations:** a FHI 360, Durham, NC, United States; b Profamilia, Biomedical Research Department, Santo Domingo, Dominican Republic

**Keywords:** Contraception, Depot medroxyprogesterone acetate, Hypopigmentation, Local tolerability, Safety, Skin discoloration, Subcutaneous

## Abstract

**Objectives:**

This study aimed to evaluate and compare local tolerability of investigational drug TV-46046 and reference drug Depo-subQ Provera 104, both containing medroxyprogesterone acetate (MPA) as an active ingredient.

**Study design:**

We conducted a randomized, crossover, single-center study. Twenty-seven healthy women aged 25 to 47 years at low risk of pregnancy received a subcutaneous injection of each of the four study drugs (120 mg/0.3 mL of TV-46046, 60 mg/0.3 mL of diluted TV-46046, 0.3 mL of TV-46046 placebo, and 104 mg/0.65 mL of Depo-subQ 104) in different quadrants of the abdomen. We assessed local tolerability by occurrence of injection site reactions (ISRs), as well as injection site pain and overall safety for at least 9 months postinjections.

**Results:**

Of a total of 108 study injections, three injections were partial due to needle blockage. We observed a total of 30 ISRs following 105 full-dose injections, including hypopigmentation (*n* = 24), bruising (*n* = 4), and atrophy/dimple (*n* = 2). Eleven cases of hypopigmentation occurred following 25 full-dose injections of undiluted TV-46046 (44.0%), six following 27 full-dose injections of diluted TV-46046 (22.2%), and seven following 26 full-dose injections of Depo-subQ 104 (26.9%). Hypopigmentations occurred on average 8 months postinjection. Injection pain was minimal and dissipated quickly after all four injections.

**Conclusions:**

Subcutaneous administration of MPA in a suspension formulation is associated with the delayed onset of hypopigmentation at the site of injection. Although not statistically significant, the rate of ISRs was over 60% higher for undiluted TV-46046 compared to Depo-subQ 104. This difference bears careful monitoring in future studies of TV-46046.

**Implications:**

From a safety standpoint, investigational drug TV-46046 is appropriate for further clinical testing as a 6-month contraceptive injectable. The previously underreported hypopigmentation associated with subcutaneous administration of MPA warrants further investigation and acceptability assessment among users of existing Depo-subQ 104 as well as careful monitoring of local tolerability of TV-46046 in future clinical trials.

**Trial registration:**

Registered at clinicaltrials.gov no: NCT02817464

## 1 Introduction

Injectable contraceptives have been safe and effective contraceptive options for decades and are used by at least 74 million women of reproductive age worldwide [1]. Depo-Provera CI (medroxyprogesterone acetate [MPA] injectable suspension, 150 mg/mL) for intramuscular use and Depo-subQ Provera 104 (104 mg/0.65 mL) for subcutaneous (SC) use (including its presentation in a prefilled syringe and in the Uniject device [Sayana Press], both referred hereafter as Depo-subQ 104) are administered on a 3-month schedule and are the most prevalent injectable methods. Relatively long intervals between injections are one of the reasons women opt for injectable contraceptives [2], and even longer intervals (e.g., 6 months) may be attractive to many potential users [3].

FHI 360 and Teva Pharmaceutical Industries Ltd. (Tel Aviv-Yafo, Israel) collaborated to develop TV-46046, a new MPA-based SC injectable that would provide contraceptive protection if injected every 6 months. The first-in-human noncomparative study (NCT02817464) suggested that TV-46046 may be associated with a higher rate of injection site reactions (ISRs) when compared to the historical data for the reference Depo-subQ 104 [4], [5]. However, the value of these data was limited due to the nondirect nature of the comparison, the small sample size of the first-in-human TV-46046 study, and potential differences in the ascertainment of local irritation. We conducted this comparative study to assess and directly compare local tolerability between TV-46046 and Depo-subQ 104.

## 2 Materials and methods

### 2.1 Study design

We conducted this randomized, single-group, crossover, local tolerability study at the Biomedical Research Department at PROFAMILIA (Santo Domingo, Dominican Republic [DR]) between December 2018 and October 2020. FHI 360′s Protection of Human Subjects Committee, the Comité de Ética de PROFAMILIA, and CONABIOS in the DR approved the study. All participants provided written informed consent before entering the study.

Healthy women aged between 18 and 50 years at low risk of pregnancy, who had no desire to become pregnant within the subsequent 18 months and had no skin disorders were eligible for the study. We excluded women who recently used contraceptive injectables, had medical contraindications or known sensitivity to MPA, or used pain medication on a chronic basis.

We hypothesized that the dose and/or concentration of MPA or the excipients of TV-46046 could be associated with local irritation. Therefore, we tested two doses and concentrations of TV-46046 400 mg/mL (120 mg/0.3 mL and 60 mg/0.3 mL of 1:1 saline-diluted TV-46046) and a single dose of TV-46046 placebo, which had an identical composition to undiluted TV-46046 but without MPA. Our reference drug was Depo-subQ 104 (0.65 mL of a 160 mg/mL formulation) supplied in a prefilled glass syringe.

We planned to enroll and randomize 24 participants to 24 injection sequences of the four study drugs. We designed the randomization scheme to result in each drug being injected first, second, third, and fourth on six occasions, and each drug being injected in each quadrant of the abdomen on six occasions. Each woman served as her own control to minimize the variability of treatment comparisons. We concealed allocation assignments using a centralized randomization application RANDOMISE within the OpenClinica data management system.

The study was not fully blinded due to differences in appearance and volume of the treatments. Designated unblinded study staff conducted randomization procedures; the staff preparing and administering injections were also unblinded to treatment sequence but were trained to shield the syringe prior to and at the time of injection from the view of the participant and study staff assessing study outcomes. Staff involved in randomization and administering injections were not involved in the assessment of study outcomes. Research staff responsible for assessing primary and secondary outcomes remained blinded to treatment assignments throughout the study.

### 2.2 Study procedures

During their enrollment visit (day 0), each participant received a total of four injections, one of each of the four study drugs: 120 mg/0.3 mL of TV-46046 (with 23 G needle), 60 mg/0.3 mL of 1:1 saline-diluted TV-46046 (with 23 G needle), 0.3 mL of TV-46046 placebo (with 23 G needle), and 104 mg/0.65 mL of Depo-subQ 104 (with the supplied 26 G needle). Each injection was separated by approximately 1 hour to eliminate carryover effect of pain of the previous injection. We administered all four injections subcutaneously in four abdominal quadrants, starting with the participant’s right upper quadrant and progressing to the left upper quadrant, left lower quadrant, and right lower quadrant per the randomized sequence. We marked each injection site at the time of the injection and, after the fourth injection, took a photo of the four marked injection sites for future identification.

We followed participants for at least 9 months after the administration of study drugs. We assessed local tolerability by evaluating ISRs twice on the day of the study drug injections (day 0): immediately after (i.e., as soon as possible but no later than 10 minutes upon removing the needle) and 1 hour (±5 minutes) after each injection; at days 1, 3, 7, and 14; at months 3 and 6; at study exit; and at additional visits if indicated. Participants with ISR(s) were followed monthly through the resolution of the ISR or month 18, whichever came first. We asked participants to assess their injection site pain twice on day 0 at the same time points when we evaluated ISRs. Thereafter, injection site pain was documented only if reported by the participants.

The scope of the safety evaluation in this study consisted of adverse events (AEs), use of concomitant medications, vital signs, and weight, measured in all participants throughout the study.

### 2.3 Outcomes

We assessed local tolerability by occurrence of ISRs, the primary outcome, that included but was not limited to erythema (i.e., redness), swelling, pruritus (i.e., itching), bleeding, bruising, injection site discoloration/hypopigmentation (i.e., lightening of the skin), or atrophy (i.e., dimple). We assessed ISRs by visual examination of the site of injection by blinded study staff and/or participant’s self-reports. In addition to documenting all ISRs, we recorded the following ISRs as AEs: injection site pain with a Numeric Rating Scale score of 7 to 10; localized injection site pruritus (or itching) requiring ≥48-hour treatment or generalized itching causing inability to perform usual social and functional activities; injection site erythema and/or induration/swelling, both of ≥5 cm in diameter (or ≥25 cm^2^ surface area) or causing greater than minimal interference with usual social and functional activities; and any ISR that met the definition of a “serious adverse event.”

For the purpose of this protocol, injection site pain, the secondary outcome, included pain associated with insertion of the needle, injecting of the drug, and any pain or tenderness at/around the injection site. Participants used an 11-point Numeric Rating Scale to assess their injection site pain by indicating a number between 0 (no pain) and 10 (worst pain). In addition, 1 hour after the fourth (final) injection, participants provided an overall ranking of all four administrations from least [1] to most [4] painful.

### 2.4 Statistical analysis

We selected the planned sample size of 24 participants to provide at least 80% power to detect differences in the rates of ISRs of the magnitude (>40%) observed in previous noncomparative studies of TV-46046 and Depo-subQ 104 (unpublished data). Although not designed to obtain precise estimates of less frequent ISR occurrence (e.g., injection site discoloration [hypopigmentation]), there was a high (>90%) probability of detecting one or more specific ISR types if the true rate of such events was at least 10%.

We summarized demographics and baseline characteristics descriptively. We summarized ISRs, the primary outcome of the study, by reaction type and treatment group and assessed pair-wise differences between treatment groups using exact McNemar’s tests conducted at the 0.05 level of significance without adjustment for multiple comparisons. We summarized injection site pain scores, the secondary outcome of the study, immediately after injection and 1 hour after injection by treatment group using descriptive statistics. We assessed differences between treatment groups in injection site pain scores immediately after injection using Poisson regression and 1 hour after injection using binomial regression. We assessed differences in ranking of the most painful injection using an exact multinomial test for homogeneity across groups. We conducted all analyses using SAS/STAT software, Version 9.4 of the SAS System for Windows (SAS Institute Inc., Cary, NC).

## 3 Results

### 3.1 Demographic and baseline characteristics

We screened 29 and enrolled 27 women in the study (93.1%). The median age of enrolled participants was 36 years, and the median weight was 66.0 kg. The majority were married/cohabitating (77.8%), had >9 full years of education (63.0%), and were biracial (and of European and African descent; 96.3%; Table 1).

**Table 1:** Baseline demographic data (treated population) in the study evaluating and comparing local tolerability following subcutaneous administration of TV-46046 and Depo-subQ Provera 104 that was conducted at the Biomedical Research Department at PROFAMILIA (Santo Domingo, Dominican Republic) between December 2018 and October 2020

Characteristic	Values (*N* = 27)
Age (y)	
Mean (SD)	35.7 (6.20)
Median (range)	36 (25-47)
Marital status, *n* (%)	
Married/cohabiting	21 (77.8)
Single	5 (18.5)
Divorced	0 (0.0)
Other	1 (3.7)
Full years of education	
Mean (SD)	9.9 (2.28)
Median (range)	10 (4-14)
Race, *n* (%)	
Biracial	26 (96.3)
Black	1 (3.7)
White	0 (0.0)
Other	0 (0.0)

### 3.2 Extent of exposure

All 27 enrolled participants received injections of all four study treatments, resulting in a total of 108 injections. All 27 participants completed the study, contributing a total of 350.4 women-months of follow-up. The final disposition is presented in Table 2.

**Table 2:** Subject disposition in the study evaluating and comparing local tolerability following subcutaneous administration of TV-46046 and Depo-subQ Provera 104 that was conducted at the Biomedical Research Department at PROFAMILIA (Santo Domingo, Dominican Republic) between December 2018 and October 2020

Disposition category	Overall	TV-46046 undiluted	TV-46046 diluted	TV-46046 placebo	Depo-subQ 104
Screened (*n*)	29				
Screening failure, *n* (%)	2 (6.9)				
Randomized (*n*)	27				
Received at least one injection, *n* (%)	27 (100.0)	27 (100.0)	27 (100.0)	27 (100.0)	27 (100.0)
Received all four injections, *n* (%)	27 (100.0)	27 (100.0)	27 (100.0)	27 (100.0)	27 (100.0)
Had a needle blockage/manipulation,^a^*n* (%)	5 (18.5)	5 (18.5)	0 (0.0)	0 (0.0)	1 (3.7)
Received partial injection,^b^*n* (%)	3 (11.1)	2 (7.4)	0 (0.0)	0 (0.0)	1 (3.7)
Total women-months of follow-up	350.4				
Completed final visit, *n* (%)	27 (100.0)				
Discontinued early, *n* (%)	0 (0.0)				

a Five participants with one or more needle blockage/manipulation were excluded from the injection site pain analysis.

b Three partial injections were excluded from ISR analysis.

Five participants experienced a total of six needle blockage events. In each instance, study staff attempted to manipulate the needle (rotate or pull) to deliver the full dose. Despite the manipulation, three participants did not receive a full dose of a study treatment (two injections of undiluted TV-46046 and one injection of Depo-subQ 104). Based on a blind review of data, the Study Review Committee overseeing this study decided to exclude the three partial injections from the primary analysis. As a result, a total of 105 full-dose injections contributed to the primary analysis (25, 27, 27, and 26 injections of undiluted TV-46046, diluted TV-46046, TV-46046 placebo, and Depo-subQ 104, respectively). All five participants who experienced needle blockage, regardless of whether the full dose was delivered, were entirely excluded from the secondary comparative pain score analysis. We expanded the final sample size from the planned 24 to 27 participants to maintain planned power when accounting for these exclusions.

### 3.3 Injection site reactions

We observed a total of 30 ISRs following 105 full-dose study injections. Fourteen of the 27 participants developed at least one ISR during the study. The differences in the rates of ISRs among the three active treatments were not significant (*p* > 0.125 for each active group comparison). However, the rate was significantly higher for each active treatment compared to TV-46046 placebo, the only group that had no ISRs (Table 3).

**Table 3:** Summary of injection site reactions by treatment group^a^ in the study evaluating and comparing local tolerability following subcutaneous administration of TV-46046 and Depo-subQ Provera 104 that was conducted at the Biomedical Research Department at PROFAMILIA (Santo Domingo, Dominican Republic) between December 2018 and October 2020

ISRs	TV-46046 undiluted (*n* = 25)	TV-46046 diluted (*n* = 27)	TV-46046 placebo (*n* = 27)	Depo-subQ 104 (*n* = 26)	Significant group differences^b^
ISRs, *n*	14	6	0	10	AC (0.000), BC (0.031), CD (0.008)
Full-dose injections with any ISRs, *n* (%)	12 (48.0)	6 (22.2)	0 (0.0)	8 (30.8)	
Full-dose injections with bruising, *n* (%)	2 (8.0)	0 (0.0)	0 (0.0)	2 (7.7)	
Full-dose injections with skin discoloration,^c^*n* (%)	11 (44.0)	6 (22.2)	0 (0.0)	7 (26.9)	AC (0.001), BC (0.031), CD (0.016)
Full-dose injections with atrophy, *n* (%)	1 (4.0)	0 (0.0)	0 (0.0)	1 (3.8)	
Full-dose injections with any other ISR, *n* (%)	0 (0.0)	0 (0.0)	0 (0.0)	0 (0.0)	


ISRs, injection site reactions.

a All 27 participants received each of the four study injections. After excluding three partial injections, 25, 27, 27, and 26 participants who received full-dose injections of undiluted TV-46046, diluted TV-46046, TV-46046 placebo, and Depo-subQ 104, respectively, contributed to the primary analysis (number of full-dose injections = number of participants in each treatment group).

b Group pairings that are significantly different (*p*-value based on McNemar’s test). A = TV-46046 undiluted, B = TV-46046 diluted, C = TV-46046 placebo, D = Depo-subQ 104.d All skin discolorations in the study were hypopigmentations.

c All skin discolorations in the study were hypopigmentations.

**Fig. 1:**
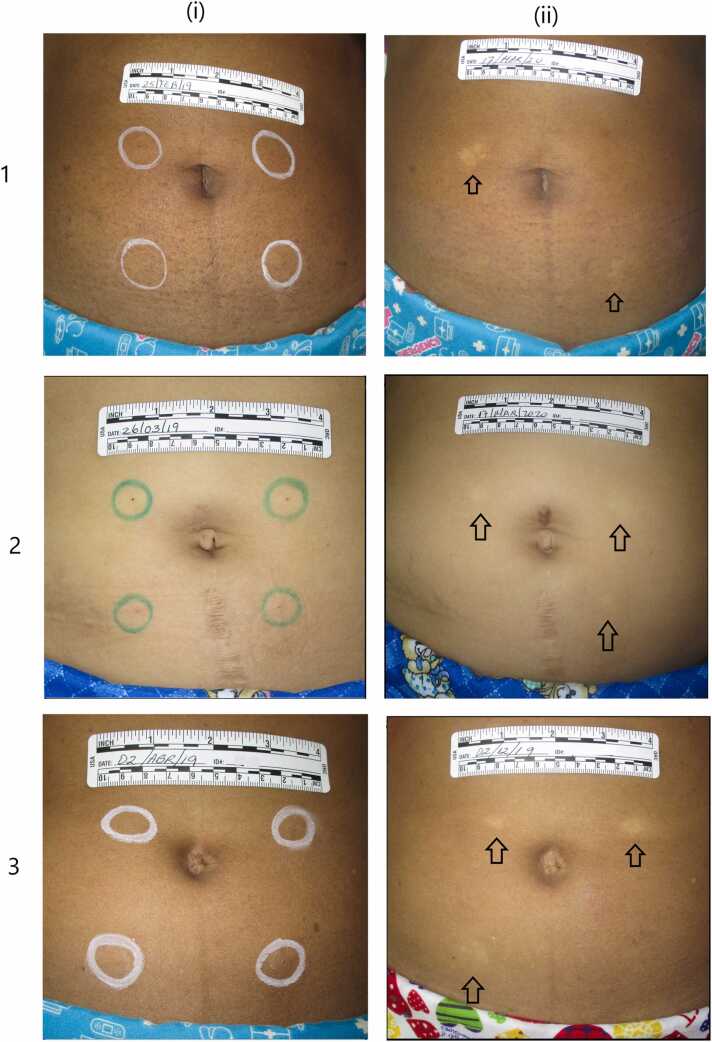
Photographs of injection sites taken on the day of injection (i) and during follow-up (ii) in the study evaluating and comparing local tolerability following subcutaneous administration of TV-46046 and Depo-subQ Provera 104 that was conducted at the Biomedical Research Department at PROFAMILIA (Santo Domingo, Dominican Republic) between December 2018 and October 2020. Participant 1: areas of hypopigmentation during follow-up at injection sites A and C; participant 2: areas of hypopigmentation during follow-up at injection sites A, B, and C; participant 3: areas of hypopigmentation during follow-up at injection sites A, B, and D.

Most of the reported ISRs (*n* = 24/30; 80%) were skin discolorations (all hypopigmentations). Of these 24 events, 11 occurred following 25 full-dose injections of undiluted TV-46046 (44.0%), six following 27 full-dose injections of diluted TV-46046 (22.2%), and seven following 26 full-dose injections of Depo-subQ 104 (26.9%). The mean diameter of hypopigmentations was 13 mm (range 5-20 mm) and similar between the treatments (Fig. 1). Approximately half of the skin discoloration ISRs were noticed and reported by study participants (45%, 50%, and 57% in the TV-46046 undiluted, TV 46046 diluted, and Depo-subQ 104 groups, respectively), while the rest were detected by study staff. On average, hypopigmentations occurred 8 months after study drug administration. Fifteen of 24 hypopigmentations (63%) resolved on or before the final visit, 18 months after dose administration (Table 4). Among those that resolved, it took an average of 6 months.

**Table 4:** Time to initiation and resolution of hypopigmentation events by treatment group in the study evaluating and comparing local tolerability following subcutaneous administration of TV-46046 and Depo-subQ Provera 104 that was conducted at the Biomedical Research Department at PROFAMILIA (Santo Domingo, Dominican Republic) between December 2018 and October 2020

Time to initiation and resolution	TV-46046 undiluted (*n* = 11)	TV-46046 diluted (*n* = 6)	Depo-subQ 104 (*n* = 7)	Overall (*n* = 24)
Time to initiation in months, mean (range)	8.2 (4.6-11.0)	7.9 (4.6-14.2)	9.0 (4.6-11.0)	8.4 (4.6-14.2)
Resolved hypopigmentations, *n* (%)	6 (55)	4 (67)	5 (71)	15 (63)
Time to resolution in months,^a^ mean (range)	6.2 (3.0-8.8)	6.3 (4.7-8.8)	5.7 (3.1-9.6)	6.1 (3.0-9.6)

a Calculation of time to resolution excluded unresolved ISRs.

Additionally, few participants reported bruising (two participants each after a complete injection of TV-46046 undiluted and Depo-subQ 104) or atrophy/dimple (one participant each in the TV-46046 undiluted and Depo-subQ 104 groups). No ISRs met the protocol definition of an AE.

### 3.4 Injection site pain

Among 22 participants with evaluable data for all four injections, mean injection site pain (SD) scores immediately after injection were 2.1 (1.72), 1.9 (1.46), 3.3 (2.15), and 1.2 (1.40) for the TV-46046 undiluted, TV-46046 diluted, TV-46046 placebo, and Depo-subQ 104 treatments, respectively. While the differences between the three active treatments were not statistically significant, they were significant for each active treatment when compared to TV-46046 placebo (*p* = 0.006, 0.014, and 0.000 for the TV-46046 undiluted, TV-46046 diluted, and Depo-subQ 104 treatments, respectively). Injection site pain was immediate and dissipated quickly as indicated by the mean (SD) scores 1 hour after the injection of 0.2 (0.50), 0.2 (0.50), 0.6 (1.00), and 0.1 (0.29) for the TV-46046 undiluted, TV-46046 diluted, TV-46046 placebo, and Depo-subQ 104 treatments, respectively (Table 5). Over half of all participants (54.5%) ranked their TV-46046 placebo injection as most painful, while only 4.5% of participants ranked their Depo-subQ 104 injection as such. The injection of undiluted and diluted TV-46046 was ranked as most painful by 22.7% and 18.2% of participants, respectively.

**Table 5:** Injection site pain scores on the day of injection by treatment group^a^ in the study evaluating and comparing local tolerability following subcutaneous administration of TV-46046 and Depo-subQ Provera 104 that was conducted at the Biomedical Research Department at PROFAMILIA (Santo Domingo, Dominican Republic) between December 2018 and October 2020

Pain score	TV-46046 undiluted (*n* = 22)	TV-46046 diluted (*n* = 22)	TV-46046 placebo (*n* = 22)	Depo-subQ 104 (*n* = 22)		Significant group differences^b^
Immediately after injection						
Mean (SD)	2.1 (1.72)	1.9 (1.46)	3.3 (2.15)	1.2 (1.40)		AC (0.006), BC (0.014), CD (0.000)
Median (range)	1.5 (0-6)	1.5 (0-5)	3.0 (0-7)	1.0 (0-5)		
≥7, *n* (%)	0 (0.0)	0 (0.0)	1 (4.5)	0 (0.0)		
1 h after injection						
Mean (SD)	0.2 (0.50)	0.2 (0.50)	0.6 (1.00)	0.1 (0.29)		AC (0.039), CD (0.017)
Median (range)	0.0 (0-2)	0.0 (0-2)	0.0 (0-3)	0.0 (0-1)		
≥7, *n* (%)	0 (0.0)	0 (0.0)	0 (0.0)	0 (0.0)		


a Analysis of injection site pain excluded five participants who experienced needle blockage/manipulation in at least one injection.

b Group pairings that are significantly different, where A = TV-46046 undiluted, B = TV-46046 diluted, C = TV-46046 placebo, D = Depo-subQ 104. *p*-values for pain immediately after injection and 1 h after injection based on Poisson regression and binomial regression, respectively.

### 3.5 General safety

Most participants (85.2%) reported at least one AE, many of which were treatment related (55.6%) but could not be attributed to a particular injection. The most frequently occurring AE in all treatment groups was headache. Only one serious AE of cholelithiasis (that was also severe) was reported during the study. It was considered not related to treatment and resolved without sequelae. No deaths or AEs leading to withdrawal occurred during the study.

## 4 Discussion

Although not statistically significant, the rate of ISRs was over 60% higher following SC administration of undiluted TV-46046 compared to Depo-subQ 104. This difference bears careful monitoring in future studies of TV-46046. The most common ISR in our study was mild skin discoloration, specifically hypopigmentation, which occurred in each of the active (i.e., MPA containing) treatments but not in TV-46046 placebo, suggesting that MPA is likely responsible for the effect. This hypothesis is supported by the findings from in vitro studies that progesterone can inhibit proliferation of human melanocytes [6] and decrease pigment production [7].

The trend toward a higher number of hypopigmentations associated with undiluted TV-46046, which contained both the highest concentration and dose of MPA, further suggests that the skin discoloration effect of MPA may be dose- or concentration-dependent, or both. Importantly, 38% of hypopigmentations did not resolve within 18 months of follow-up, and for those that did resolve, it took on average 6 months.

Our findings are consistent with the results from a recently completed multicenter phase 3 trial of Sayana Press when injected every 4 months [8], which also reported delayed onset of hypopigmentation at the site of injection. Of all MPA-containing injectable products, only Depo-Provera for intramuscular use (MPA injectable suspension; 400 mg/mL) has change in the color of skin as an adverse reaction on its label [9]. There are several possible explanations of why hypopigmentation was not reported previously for Depo-subQ 104. Due to its delayed nature and relatively small size, this reaction may not be noticed, reported, or associated with the injection by providers or users. In our study, only half of skin discolorations were noticed by study participants. Race may be another reason: with 96.3% and 62% of study participants being biracial in this and the Sayana Press study, respectively; it is possible that darker skin tones make hypopigmentation more noticeable. In addition, injection sites were evaluated for possible reactions systematically throughout both studies, so the higher rate of ISRs compared to the prior data may also be due to the ascertainment bias.

Of the four study injections, TV-46046 placebo was the most painful, and Depo-subQ 104 was the least painful. Importantly, injection pain in all groups was minimal and short lived. While injection site pain, given its mild and transient nature, is unlikely to have a major impact on acceptability of the new injectable, future trials should evaluate and provide more conclusive evidence of acceptability and satisfaction with TV-46046.

Our study has both strengths and weaknesses. The main strength of our study is its crossover design, which enabled us to test all four study drugs with women serving as their own control. Randomization to 24 unique injection sequences of the four study drugs counterbalanced the potential effects of injection order or abdominal quadrant on study outcomes. Other strengths included the extended follow-up period, allowing us to identify late-onset ISRs that could have been missed in other studies, as well as to assess the time to their resolution. Ethnic homogeneity of our study population is a possible weakness. With more than 96% of study participants being biracial with dark skin, generalizability of our results may be limited. In addition, the reference drug was administered with the needle of a smaller diameter compared with the investigational drug, which may explain lesser pain reported in that group.

Based on our findings, we conclude that from the safety standpoint, investigational TV-46046 is appropriate for further clinical testing as a 6-month contraceptive injectable. We also demonstrated that the SC use of MPA is associated with the delayed onset of hypopigmentation at the injection site. While it is not a safety concern and size of skin discolorations is relatively small, its long-term nature may be problematic, especially for users with dark skin. Multiple re-injections, particularly in exposed areas, such as the upper arm, may be considered an undesirable cosmetic defect and adversely impact the acceptability. This previously underreported finding requires further research and acceptability assessment among users of existing Depo-subQ 104 as well as careful monitoring of local tolerability of TV-46046 in future clinical trials.

## Declaration of Competing Interest

The authors declare that they have no known competing financial interests or personal relationships that could have appeared to influence the work reported in this article.
