# Supplementary material for: A randomized crossover study to evaluate local tolerability following subcutaneous administration of a new depot medroxyprogesterone acetate contraceptive formulation
Source: Contracept X. 2023 Sep 12;5:100100. doi: 10.1016/j.conx.2023.100100 (PMC10562734; doi:10.1016/j.conx.2023.100100)
Supplement: Supplementary file 1 — Supplementary material [file mmc1.pdf]

Clinical Study Protocol with Amendment 04

Study Number TV46046-WH-10147

FHI 360 Study Number: 1191167

A Randomized Crossover Study to Evaluate Local Tolerability Following Subcutaneous Administration of TV-46046

Phase 1

IND number: 126249

Amendment 04 Approval Date: 18 May 2020

Amendment 03 Approval Date: 19 November 2019

Amendment 02 Approval Date: 11 April 2019

Amendment 01 Approval Date: 02 July 2018

Original Protocol Approval Date: 15 May 2018

Sponsor
Teva Branded Pharmaceutical
Products R&D, Inc.
145 Brandywine Pkwy,
West Chester, PA 19380
United States

Monitor
FHI 360
359 Blackwell Street, Suite 200
Durham, North Carolina 27701
United States

Authorized Representative
Alexandra Kropotova, MD, MBA
Vice President, Global Specialty R&D, Respiratory TA Head
Teva Branded Pharmaceutical Products R&D, Inc.
145 Brandywine Pkwy,
West Chester, PA 19380
United States
+1-610-738-6557

Sponsor's Medical Expert
Michael J. Lamson, PhD
VP, PK and ClinPharm
Nuventra, Inc.™
2525 Meridian Parkway, Suite 280
Durham, North Carolina 27713
+1-919-667-7976

Sponsor's Safety Representative
Dana Ilic Bogojevic, MD
Safety Physician, Medical Scientific Unit – US
Teva Branded Pharmaceutical Products R&D, Inc.
400 Interpace Pkwy, Bldg. A, Suite 367
Parsippany, NJ 07054
+1-973-658-0378

Information regarding other departments and institutions is found in Appendix A.

This clinical study will be conducted in accordance with current Good Clinical Practice (GCP) as directed by the provisions of the International Council for Harmonisation (ICH); United States (US) Code of Federal Regulations (CFR), and European Union (EU) Directives and Regulations (as applicable in the region of the study); national country legislation; and the sponsor and FHI 360's Standard Operating Procedures (SOPs).

Confidentiality Statement

This document contains confidential and proprietary information (including confidential commercial information pursuant to 21CFR§20.61) and is a confidential communication of Teva Branded Pharmaceutical Products R&D, Inc. and/or its affiliates. The recipient agrees that no information contained herein may be published or disclosed without written approval from the sponsor.

© 2020 Teva Branded Pharmaceutical Products R&D, Inc. All rights reserved.

AMENDMENT HISTORY

The protocol for Study TV46046-WH-10147 (original protocol dated 15 May 2018) has been amended and reissued as follows:

Amendment 04	18 May 2020<br>27 subjects have been enrolled to date
Amendment 03	19 November 2019<br>27 subjects have been enrolled to date.
Amendment 02	11 April 2019<br>13 subjects have been enrolled to date.
Amendment 01	02 July 2018<br>No subjects have been enrolled to date.

Details about the changes and reason/justification for each change are provided in Section 16.

INVESTIGATOR AGREEMENT

Clinical Study Protocol with Amendment 04

Original Protocol Dated 15 May 2018

Study Number TV-46046-WH-10147

FHI 360 Study Number: 1191167

IND number: 126249

A Randomized Crossover Study to Evaluate Local Tolerability Following Subcutaneous Administration of TV-46046

Investigator: Vivian Brache, Lic

Title: Director of the Biomedical Research Department

Address of Investigational Center: Asociación Dominicana
Pro Bienestar de la Familia, Inc. (PROFAMILIA)
Nicolas de Ovando esq. Calle 16 Ens. Luperon
Santo Domingo, Dominican Republic

Tel: 1-809-681-8357

I have read the protocol with Amendment 04 and agree that it contains all necessary details for carrying out this study. I am qualified by education, experience, and training to conduct this clinical research study. The signature below constitutes agreement with this protocol and attachments, and provides assurance that this study will be conducted according to all stipulations of the protocol, including all statements regarding confidentiality, and according to national or local legal and regulatory requirements and applicable regulations and guidelines.

I will make available the protocol and all information on the investigational medicinal product (IMP) that were furnished to me by the sponsor to all physicians and other study personnel reporting to me who participate in this study and will discuss this material with them to ensure that they are fully informed regarding the IMP and the conduct of the study. I agree to keep records on all subject information, investigational medicinal products (IMP) shipment and return forms, and all other information collected during the study, in accordance with national and local Good Clinical Practice (GCP) regulations as well as all other national and international laws and regulations.

**Investigator**	**Signature**	**Date**

SPONSOR PROTOCOL APPROVAL

**Sponsor's Authorized Representative**	**Signature**	**Date**
Alexandra Kropotova MD, MBA<br>Vice President, Global Specialty R&D, Respiratory TA Head	[illegible]	5-20-2020

CLINICAL STUDY PROTOCOL SYNOPSIS

With Amendment 04

Study: TV46046-WH-10147

Title of Study: A Randomized Cross-over Study to Evaluate Local Tolerability Following Subcutaneous Administration of TV-46046

Sponsor: Teva Branded Pharmaceutical Products R&D, Inc.

Investigational New Drug (IND) Number: 126249

EMA Decision number of Pediatric Investigation Plan: Article 45 or 46 of 1901/2006 does not apply

Name of Test Investigational Medicinal Product (IMP): Depot Medroxyprogesterone Acetate (DMPA [TV-46046])

Active Substance: Medroxyprogesterone acetate (MPA)

EudraVigilance (EV) code for the IMP, if applicable: NA

Type of Study: Safety/Tolerability

Phase of Clinical Development: 1

Number of Investigational Centers Planned: 1

Countries Planned: Dominican Republic

Planned Study Period: The study is expected to start in Q3 2018 and have a duration period of approximately 24 months including approximately 6 months of recruitment and up to 18 months of follow-up.

Primary Objective(s): The primary objective is to evaluate and compare local tolerability associated with subcutaneous administration of 120 mg/0.3 mL of TV-46046, 60 mg/0.3 mL of 1:1 saline-diluted TV-46046, 0.3 mL of TV-46046 Placebo, and 104 mg/0.65 mL of Depo-subQ Provera 104® (medroxyprogesterone acetate injectable suspension, 104 mg/0.65 mL, hereafter referred to as Depo-subQ 104).

Secondary Objective(s): The secondary objectives are:

1. To evaluate and compare injection site pain associated with subcutaneous administration of 120 mg/0.3 mL of TV-46046, 60 mg/0.3 mL of 1:1 saline-diluted TV-46046, 0.3 mL of TV-46046 Placebo, and 104 mg/0.65 mL of Depo-subQ 104
2. To evaluate the overall safety of subcutaneous same-day administration of 120 mg/0.3 mL of TV-46046, 60 mg/0.3 mL of 1:1 saline diluted TV-46046, 0.3 mL of TV-46046 Placebo, and 104 mg/0.65 mL of Depo-subQ 104

Exploratory and Other Objectives: NA

General Study Design and Methodology:

This is a randomized, crossover, single-center, Phase 1 study to evaluate local tolerability following subcutaneous administration of TV-46046 in 24 healthy female subjects 18 to 50 years of age. After signing the informed consent form, eligible subjects will be enrolled in the study and receive a subcutaneous injection of each of the four study drugs: 120 mg/0.3 mL of

TV-46046, 60 mg/0.3 mL of 1:1 saline-diluted TV-46046, 0.3 mL of TV-46046 Placebo, and 104 mg/0.65 mL of Depo-subQ 104. Injections will be administered in different quadrants of the abdomen, each separated by approximately 1 hour. Subjects will be randomized to one of 24 different injection sequences (one subject per sequence) to counterbalance potential effects of injection order or abdominal quadrant on study outcomes.

All subjects will be followed for at least 6 months after receiving their injections. Local tolerability will be assessed by evaluating ISRs at least twice on the day of the study drug injection (Day 0: immediately after [ie, as soon as possible but no later than 10 minutes upon removing the needle] and 1 hour [±5 minutes] after the injection), at Days 1, 3, 7, and 14; at Months 3 and 6; and at other visits, if indicated. Subjects with unresolved ISR(s) will be followed monthly through the resolution of ISR(s) or Month 18, whichever comes first. Subjects will assess their injection site pain using an 11-point Numeric Rating Scale (NRS) (0 = no pain at all; 10 = worst pain) twice on the day of injection (Day 0: immediately after [ie, as soon as possible but no later than 10 minutes upon removing the needle] and 1 hour [±5 minutes] after the injection, but prior to any subsequent injection). In addition, 1 hour after the fourth (final) injection subjects will rank administrations from least [1] to most [4] painful. Thereafter, injection site pain will be assessed by self-reports at Days 1, 3, 7, and 14; at Months 3 and 6; and other visits, if indicated. Adverse events and concomitant medications will be recorded throughout the study. Vital signs and weight will be measured at baseline, Month 6, and at study exit.

In the ongoing TV46046-WH-10147 study, several cases of hypopigmentation had a later onset (ie, approximately 6 months post-injection) than observed in Study TV46046-WH-10075. In order to identify any late onset ISRs, subjects who have exited the study at Month 6 with no new or ongoing ISRs and who agree to return for a single follow-up visit between Months 9 and 12, will undergo ISR, adverse event, and concomitant medication evaluation, and vital sign and weight measurements at that time.

Investigational Medicinal Products (IMPs): Dose, Pharmaceutical Form, Route of Administration, and Administration Rate

Test IMP: DMPA (120 mg/0.3 mL of TV-46046)

Reference IMP: Medroxyprogesterone acetate injectable suspension (104 mg/0.65 mL)

Placebo IMP: TV-46046 Placebo (vehicle only)

Study Population and Number of Subjects Planned: Approximately 24 women will be enrolled. Subjects will not be replaced in this study. Subjects may be added if more than 2 women fail to complete the treatment sequence or discontinue before Month 6.

Duration of Subject Participation: Total duration of the study for each subject is expected to be 6 to 18 months including 1 day for screening, enrollment and administration of treatment, and 6 to 18 months of follow-up.

Main Criteria for Inclusion: Subjects may be included in the study if they meet all of the following criteria:

a. has a low risk of pregnancy (ie, sterilized, in exclusively same-sex partnership, in menopause and/or post-menopausal, abstinent, in monogamous relationship with vasectomized partner, using nonhormonal intrauterine device (IUD) or consistent use of condoms)

b. is in good general health as determined by a medical history
c. is 18 to 50 years of age (inclusive)
d. is willing to provide informed consent and follow all study requirements
e. is not pregnant and does not have desire to become pregnant in the subsequent 18 months
f. had a normal mammogram within the last year, if 40 years or older
g. has no skin disorders or skin allergies

Main Criteria for Exclusion: Subjects will be excluded from participating in this study if they meet any of the following criteria:

a. has hypertension,
    - systolic blood pressure (BP) ≥160 mm Hg or diastolic BP ≥100 mm Hg or
    - vascular disease
b. has ischemic heart disease or a history of ischemic heart disease
c. has a history of stroke
d. has a history of thromboembolic event (s)
e. has systemic lupus erythematosus or
    - positive (or unknown) antiphospholipid antibodies or
    - severe thrombocytopenia
f. has rheumatoid arthritis and is undergoing immunosuppressive therapy
g. has migraine with aura
h. has unexplained vaginal bleeding
i. has diabetes
j. has a strong family history of breast cancer (defined as one or more first degree relatives, breast cancer occurring before menopause in three or more family members, regardless of degree of relationship, or any male family member with breast cancer), or current or history of breast cancer, or undiagnosed mass detected by breast exam
k. has cervical cancer or a history of cervical cancer
l. has severe cirrhosis (decompensated) or liver tumors
m. has known significant renal disease
n. has a history of diagnosed clinical depression or bipolar disorder, with or without suicidal ideation, and/or history of suicide attempt
o. has, in the last two years, a history of either hospitalization or medication management for a psychiatric disorder that in the opinion of the investigator would make study participation unsafe, would interfere with adherence to study requirements or complicate data interpretation

p. is currently using hormonal contraception

q. had an injection of DMPA (Depo-Provera CI or Depo-subQ 104) in the past 12 months; or combined injectable in the last 3 months

r. has a known sensitivity to MPA or any inactive ingredients

s. is chronically using pain medication

t. has a plan to move to another location in the next 18 months

u. has, in the opinion of the investigator, a potentially elevated risk of human immunodeficiency virus (HIV) infection (eg, HIV-positive partner, intravenous (IV) drug use by self or by partner)

v. has any condition (social or medical), which in the opinion of the investigator would make study participation unsafe, would interfere with adherence to the clinical study requirements, or would complicate data interpretation

Study Evaluations

Safety and Tolerability Assessments

Local Tolerability

Local tolerability will be assessed by occurrence of ISRs including but not limited to erythema (redness), swelling, pruritus (itching), bleeding, bruising, injection site discoloration (eg, hypopigmentation), or atrophy (ie, dimple). The site of injection will be evaluated for possible ISRs after each of the four injections at least twice during the enrollment/injection visit (Day 0: immediately after (ie, as soon as possible but no later than 10 minutes upon removing the needle) and 1 hour (±5 minutes) after each injection (right before the next injection). Thereafter, injection sites will be monitored for the progress of ongoing and/or occurrence of new ISRs at Days 1, 3, 7, and 14; at Months 3 and 6, and at other visits, if indicated.

Injection Site Pain

Injection site pain will be evaluated twice on the day of the study drugs injection (Day 0): immediately after (ie, as soon as possible but no later than 10 minutes upon removing the needle) and 1 hour (±5 minutes) after each injection (right before the next injection). Subjects will assess their injection site pain using an 11-point NRS (0 = no pain at all; 10 = worst pain). In addition, 1 hour after the fourth (final) injection subjects will provide an overall ranking of all four administrations from least [1] to most [4] painful.

Thereafter, injection site pain will be evaluated by self-reports on Days 1, 3, 7, and 14, Month 3 and 6, and at additional visits, if appropriate. If the subject reports injection site pain, she will be asked to use the NRS to evaluate the intensity of pain in order to determine if it meets the definition of an adverse event.

Overall Safety

Overall safety will be assessed by occurrence of adverse events, use of concomitant medications, and changes in vital signs and weight compared to baseline.

Statistical Methods

Sample Size Determination: The study size of 24 subjects (N=24) will provide at least 80% power to detect differences in ISR rates of the magnitude (>40%) observed in recent non-comparative studies of TV-46046 and Depo-subQ 104. Although not designed to obtain precise estimates of less frequent ISR rates (eg, injection site discoloration [hypopigmentation]), there is a high (>90%) probability of detecting one or more specific ISR types if the true rate of such events is at least 10%. No randomized subjects will be replaced. However, if more than two subjects fail to receive all four injections or discontinue before Month 6, then additional subjects may be enrolled to help ensure that there are at least 22 subjects who complete the treatment sequence and are evaluable for at least 6 months.

Primary Measure:

- ISR

Secondary Measure:

- Injection site pain evaluated using NRS

Analyses

Safety Analysis: Safety and tolerability will be evaluated based on the following measures:

- ISRs
- Injection site pain
- Subjects' perception of pain
- Occurrence of adverse events
- Use of concomitant medications
- Change in vital signs and weight

TABLE OF CONTENTS

TITLE PAGE 1
AMENDMENT HISTORY 3
INVESTIGATOR AGREEMENT 4
CLINICAL STUDY PROTOCOL SYNOPSIS 5
LIST OF ABBREVIATIONS AND DEFINITIONS OF TERMS 16
1. BACKGROUND INFORMATION 18
1.1. Introduction 18
1.2. Findings from Nonclinical and Clinical Studies 18
1.2.1. Nonclinical Studies 18
1.2.2. Clinical Studies 19
1.3. Known and Potential Benefits and Risks 19
1.3.1. Overall Potential Benefits and Risks 19
1.3.2. Known and Potential Risks of TV-46046 and Depo-subQ 104 19
1.3.2.1. Pregnancy 19
1.3.2.2. Injection Site Reactions 20
1.3.2.3. Menstrual Changes 20
1.3.2.4. Weight Gain 21
1.3.2.5. Risk of Human Immunodeficiency Virus 21
1.3.2.6. Return to Fertility 21
1.3.2.7. Bone Mineral Density 22
1.3.2.8. Dose of MPA 22
1.3.2.9. Other 23
1.4. Study Design Rationale 24
1.4.1. General Study Design Rationale 24
1.4.2. Dosage Rationale 24
2. STUDY OBJECTIVES AND MEASURES/PARAMETERS 26
2.1. Study Objectives 26
2.1.1. Primary Objective 26
2.1.2. Secondary Objectives 26
2.2. Study Endpoints 26
2.2.1. Primary Endpoint 26

2.2.2. Secondary Endpoints 26
2.2.2.1. Injection Site Pain....26
2.2.2.2. Overall Safety....27
3. INVESTIGATIONAL PLAN 28
3.1. General Study Design 28
3.2. Subject Eligibility....28
3.2.1. Subject Inclusion Criteria 28
3.2.2. Subject Exclusion Criteria 29
3.3. Duration of Subject Participation....30
3.4. Study Procedures....30
3.4.1. Procedures for Screening Visit....33
3.4.2. Procedures for Enrollment/Injections Visit....33
3.4.3. Follow-up/Final Visits 34
3.4.4. Unscheduled Visits 35
3.4.5. Post-6 Month Follow-up 35
4. TREATMENT OF SUBJECTS 36
4.1. Investigational Medicinal Products Administered During the Study....36
4.1.1. Test Investigational Medicinal Product....36
4.1.2. Reference Investigational Medicinal Product 36
4.1.3. Placebo Investigational Medicinal Product....36
4.2. Treatment of Subjects....36
4.3. Blinding/Unblinding....39
4.3.1. Unblinding During the Study....39
4.4. Stopping Rules and Discontinuation Criteria....39
4.5. Interim Data Reviews 40
4.6. Prior and Concomitant Therapy or Medication....40
4.6.1. Subject Withdrawal Criteria and Procedures 41
4.7. Procedures for Monitoring Subject Compliance....41
4.8. Withdrawal of Subjects....41
4.8.1. Lost to Follow-up 41
5. SAFETY AND TOLERABIILITY MEASUREMENTS AND ASSESSMENTS 42
5.1. Safety and Tolerability Measurements 42

5.1.1. Local Tolerability 42
5.1.2. Injection Site Pain 42
5.2. Safety Assessments 43
5.2.1. Definition of an Adverse Event 43
5.2.2. Recording and Reporting Adverse Events 44
5.2.3. Severity of an Adverse Event 45
5.2.4. Relationship of an Adverse Event to the IMP 45
5.2.5. Serious Adverse Events 46
5.2.5.1. Definition of a Serious Adverse Event 46
5.2.5.2. Expectedness 47
5.2.5.3. Reporting a Serious Adverse Event 47
5.2.6. Protocol Defined Adverse Events for Expedited Reporting 48
5.2.7. Weight and Vital Signs 49
5.2.8. Medication Error and Special Situations Related to the Investigational Medicinal Products 49
5.2.9. Protocol Deviations Because of an Adverse Event 50
5.2.10. Pregnancy 50
6. PHARMACOKINETIC MEASUREMENTS AND ASSESSMENTS 51
7. PHARMACODYNAMIC, PHARMACOGENOMIC, IMMUNOGENICITY, AND BIOMARKER MEASUREMENTS AND ASSESSMENTS 52
7.1. Pharmacodynamics 52
7.2. Pharmacogenomic Assessments 52
7.3. Immunogenicity 52
7.4. Assessment of Exploratory Biomarkers 52
8. STATISTICS 53
8.1. Sample Size and Power Considerations 53
8.2. Method of Randomization 53
8.3. Analysis Sets 54
8.4. Data Handling Conventions 54
8.5. Study Population 54
8.5.1. Subject Disposition 54
8.5.2. Demographic and Baseline Characteristics 54

8.6. Analysis of Primary Endpoints 54
8.6.1. Local Tolerability 54
8.7. Analysis of Secondary Endpoints 55
8.7.1. Injection Site Pain 55
8.7.2. Overall Safety 55
9. INVESTIGATIONAL MEDICINAL PRODUCT INFORMATION 57
9.1. Investigational Medicinal Product Storage and Security 57
9.2. Investigational Medicinal Product Accountability 57
10. QUALITY CONTROL AND QUALITY ASSURANCE 58
10.1. Protocol Amendments, Protocol Deviations, and Important Protocol Deviations 58
10.1.1. Protocol Amendments 58
10.1.2. Important Protocol Deviations 58
10.2. Study Monitoring 58
10.3. Clinical Product Complaints 59
10.3.1. Product Complaint Information Needed from the Investigational Center 59
10.3.2. Handling the Product Complaint IMP at the Investigational Center 60
10.3.3. Documenting a Product Complaint 60
10.4. Data Quality Control 60
10.5. Audit and Inspection 61
11. ETHICAL AND REGULATORY CONSIDERATIONS 62
11.1. Investigator Responsibilities 62
11.2. Health Authorities and Independent Ethics Committees/Institutional Review Boards 62
11.3. Informed Consent 63
11.4. Subject Confidentiality 63
11.5. Declaration of the End of the Clinical Study 63
11.6. Registration of the Clinical Study 63
12. STUDY DOCUMENTATION 64
12.1. Source Data and Case Report Forms 64
12.2. Archiving of Study Documentation 64
12.2.1. FHI 360 Responsibilities 64
12.2.2. Investigator Responsibilities 65

13. FINANCING AND INSURANCE....66
14. REPORTING AND PUBLICATION OF RESULTS....67
15. REFERENCES....68
16. SUMMARY OF CHANGES TO PROTOCOL....70
16.1. Protocol Amendment 04 Dated 18 May 2020....70
16.2. Protocol Amendment 03 Dated 19 November 2019....72
16.3. Protocol Amendment 02 Dated 11 April 2019....78
16.4. Protocol Amendment 01 Dated 02 July 2018....79
APPENDIX A. STUDY RESPONSIBILITIES....83

LIST OF TABLES

Table 1: Study Procedures and Assessments 31
Table 2: Investigational Medicinal Products Used in the Study 38
Table 3: ISR Rates (per injection) 53

LIST OF ABBREVIATIONS AND DEFINITIONS OF TERMS

Abbreviation	Term
BMD	bone mineral density
BP	blood pressure
CDMS	clinical data management system
CFR	Code of Federal Regulations (US)
CI	Contraceptive Injection
CRF	case report form (refers to any media used to collect study data [ie, paper or electronic])
CRO	contract research organization
DSS	docusate sodium sulfosuccinate
DMPA	depot medroxyprogesterone acetate
EC	Ethics Committee
EMA	European Medicines Agency
EU	European Union
FDA	Food and Drug Administration (US)
FIH	first in human
GCP	Good Clinical Practice
HIV	human immunodeficiency virus
IB	Investigator’s Brochure
ICF	informed consent form
ICH	International Council for Harmonisation
IEC	Independent Ethics Committee
IM	intramuscular
IMP	investigational medicinal product
IRB	Institutional Review Board
ISR	injection site reaction
IUD	intrauterine device
IV	intravenous
MEC	Medical Eligibility Criteria
MPA	Medroxyprogesterone acetate
NDA	New Drug Application
NRS	Numerical Rating Scale
OTC	over-the-counter

Abbreviation	Term
PDAE	protocol defined- adverse event
PHSC	Protection of Human Subjects Committee
RSI	Reference safety information
SAE	serious adverse event
SC	subcutaneous
SOP	standard operating procedure
SPC	Summary of Product Characteristics
SRC	study review committee
SUSAR	suspected unexpected serious adverse reaction
US	United States (of America)
WHO	World Health Organization

1. BACKGROUND INFORMATION

Teva and FHI 360 are in collaboration to develop a new formulation of depot medroxyprogesterone acetate (DMPA), TV-46046, for the prevention of pregnancy when injected every 6 months.

1.1. Introduction

Teva has been conducting a Phase 1 pharmacodynamic (PD) and pharmacokinetic (PK) study to identify a dose of medroxyprogesterone acetate (MPA) injectable suspension 400 mg/mL (TV-46046) (within the range of 80 to 300 mg) that is both safe and consistent with a high degree of contraceptive efficacy when injected every 6 months (A Two-part, Phase 1, Exploratory and Dose-range Finding Study to Evaluate Suppression of Ovulation and Pharmacokinetics of Medroxyprogesterone Acetate Following a Single Subcutaneous Administration of TV-46046 in Women with Ovulatory Cycle [TV46046-WH-10075]). The interim data from Part 1 of this first in human (FIH) non-comparative study suggested that the single 120 mg dose of TV-46046 injected subcutaneously in the abdomen may be associated with a higher rate of local irritation when compared to the historical data for the reference Depo-subQ Provera 104® (hereafter referred to as Depo-subQ 104). This comparison should be interpreted with caution due to its non-direct nature, the small sample size, and the potential data collection biases of Study TV-46046-WH-10075. Teva (the sponsor) and FHI 360 propose to conduct a local tolerability study to directly evaluate and compare local tolerability and injection pain following subcutaneous administration of TV-46046 and Depo-subQ 104.

1.2. Findings from Nonclinical and Clinical Studies

1.2.1. Nonclinical Studies

The sponsor's current nonclinical safety program for TV-46046 consists of four in vivo nonclinical studies: a Good Laboratory Practice (GLP) female rabbit subcutaneous local tolerance/distant target organ tissue evaluation with long term toxicokinetic study (Study DS-2015-009); a GLP murine local lymph node assay study (Study DS-2015-017); a non-GLP murine local lymph node assay study (Study DS-2014-064); and a non-GLP female rabbit subcutaneous pharmacokinetic and gross tolerability study (Study DP-2014-135).

No significant injection site reactions (ISRs) occurred following treatment with TV-46046 in the 9-month local tolerability study in rabbits (Study DS-2015-009). Between days 43 and 90, grade 1 hypopigmentation was observed intermittently in 2 rabbits given 104 mg/0.65 mL of Depo-subQ 104, and in 3 rabbits given 300 mg/mL of TV-46046. Between days 92 and 180, hypopigmentation was observed in 2 rabbits given Depo-subQ 104 and in 5 rabbits given TV-46046. Between days 183 and 246 hypopigmentation was observed in 1 rabbit only in the TV-46046 group. There were no significant injection site differences in microscopic findings between TV-46046 and the reference Depo-subQ 104.

Based on the results of the IND-enabling nonclinical investigation, the sponsor did not anticipate the rate of ISRs associated with the subcutaneous administration of TV-46046 in humans to be

higher than that of Depo-subQ 104. However, careful evaluation of the risk of ISRs was planned in Study TV-46046-WH-10075.

1.2.2. Clinical Studies

Based on the interim analysis of safety data from the TV-46046-WH-10075 study on 28 August 2017, eleven out of 12 study subjects reported at least one sign or symptom consistent with an ISR that included but was not limited to pain, redness, tenderness, swelling, bruising, itching and sense of pressure during injection. Nine of the reported ISRs met the per protocol definition of an adverse event. All nine adverse events were mild in severity, lasted on average 4 to 5 days and resolved without sequelae. Two women had hypopigmentation of skin at the site of injection (ie, injection site discoloration) that appeared between 3 and 4 months after the injection, respectively. Both areas of hypopigmentation were approximately 2 cm in diameter and both were still present 6 months after the injection. More detailed information on the results of the Phase 1 study may be found in the current investigational brochure (IB).

1.3. Known and Potential Benefits and Risks

In addition to the benefit and risk information described in this section, please refer to the current IB (Section 3, Section 6.3, and Section 7).

1.3.1. Overall Potential Benefits and Risks

The study will be conducted in healthy subjects; no direct health benefits are expected in this study population. The study design, inclusion/exclusion criteria, and procedures have been developed in a manner to protect subject safety. The results of this study may facilitate the development of a new longer-acting injectable contraceptive.

1.3.2. Known and Potential Risks of TV-46046 and Depo-subQ 104

The contraceptive dose, as well as the duration of contraceptive effects, of TV-46046 are unknown. Therefore, all necessary precautions will be taken to minimize the risk of pregnancy among study subjects. Only women who are not pregnant, not wanting to become pregnant in the next 18 months, and who are at low risk of pregnancy because they are sterilized, in exclusively same-sex partnership, in menopause, and/or postmenopausal, abstinent, in a monogamous relationship with vasectomized partner, using a nonhormonal IUD, or consistently using condoms (see Section 3.2.1 for Inclusion Criteria) will be enrolled in this study. During the study, pregnancy testing will be performed if the woman is experiencing any symptoms or signs of pregnancy, or if she thinks she may be pregnant.

1.3.2.1. Pregnancy

Although no contraceptive injectables should be used during pregnancy, there appears to be little or no increased risk of birth defects in women who have inadvertently been exposed to MPA injections in early pregnancy. Neonates exposed to MPA in-utero and followed to adolescence showed no evidence of any adverse effects on their health including their physical, intellectual, sexual, or social development (Depo-Provera CI US Prescribing Information 2016). According to the prescribing information, women who are breastfeeding should not have their first injection until the sixth postpartum week (Depo-Provera CI US Prescribing Information 2016). However,

neonates and infants exposed to MPA from breast milk have been studied for developmental and behavioral effects through puberty, and no adverse effects have been noted. According to the Centers for Disease Control and Prevention (CDC) US Medical Eligibility Criteria (MEC) for Contraceptive Use, all progestin-only hormonal methods, including progestin-only pills, DMPA, and implants, are safe for postpartum women, including women who are breastfeeding, and can be initiated immediately postpartum (Curtis 2016).

1.3.2.2. Injection Site Reactions

In 5 clinical studies involving 2325 women using Depo-subQ 104 (282 treated for up to 6 months, 1780 treated for up to 1 year and 263 treated for up to 2 years, ie, 8 injections), 5% reported ISRs, and 1% had persistent skin changes, typically described as small areas of induration or atrophy (Depo-subQ 104 US Prescribing Information 2016). One of these studies found more injection site reactions (which were all mild to moderate in severity) in the Depo-subQ 104 (subcutaneous injection) group (8%) than in the Depo-Provera CI (intramuscular injection) group (0.4%), an expected finding for the subcutaneous route of administration (Kaunitz et al 2009).

Based on the nonclinical data, Teva did not anticipate the rate of ISRs associated with the subcutaneous administration of TV-46046 in humans to be higher than that of Depo-subQ 104 but planned for the careful evaluation of the risk of ISRs in Study TV-46046-WH-10075. The interim results from Study TV-46046-WH-10075 indicated higher rates of local irritation associated with TV-46046 (eg, pain, redness, swelling, itching) compared to the historical data for Depo-subQ 104 (Depo-subQ 104 US Prescribing Information 2016). Eleven out of 12 participants reported at least one sign or symptom consistent with an ISR that included but was not limited to pain, redness, tenderness, swelling, bruising, itching and sense of pressure during injection. Nine of the reported ISRs met the per protocol definition of an adverse event. All nine adverse events were mild in severity, lasted on average 4 to 5 days and resolved without sequelae. Two women had hypopigmentation of skin at the site of injection (ie. injection site discoloration) that appeared between 3 and 4 months after the injection, respectively. Both areas of hypopigmentation were approximately 2 cm in diameter and both were still present 6 months after the injection. Based on interim results from Study TV-46046-WH-10075, there are no safety concerns associated with the subcutaneous administration of TV-46046. However, all subjects will be fully informed of the potential risk of ISRs including injection site discoloration (hypopigmentation) before joining the study.

1.3.2.3. Menstrual Changes

Most women using Depo-Provera CI or Depo-subQ 104 experience disruption of menstrual bleeding patterns. Altered menstrual bleeding patterns include amenorrhea, irregular or unpredictable bleeding or spotting, prolonged spotting or bleeding, and heavy bleeding. As women continue using Depo-Provera CI or Depo-subQ 104, fewer experience irregular menstrual bleeding and more experience amenorrhea. In three contraception trials, 39.0% of women experienced amenorrhea during month 6, and 56.5% experienced amenorrhea during month 12 (Depo-subQ 104 US Prescribing Information 2016).

In the ongoing study (Study TV-46046-WH-10075), the effect of a single subcutaneous administration of TV-46046 on bleeding pattern was consistent with that of Depo-Provera CI or

Depo-subQ 104. At 3 months post-injection, 3 out of 6 subjects experienced spotting. At 7.5 months post-injection, 2 out of 4 subjects reported spotting, and the other 2 reported irregular bleeding. When asked about their overall opinion on the bleeding pattern, most of the women responded that it was acceptable.

All subjects will be informed before joining the local tolerability study that their bleeding pattern is likely to be irregular. Changes in menstrual bleeding patterns and its acceptability will not be evaluated.

1.3.2.4. Weight Gain

Women tend to gain weight while using both Depo-Provera CI and Depo-subQ 104. From an initial average body weight of 136 pounds, women who completed 1 year of treatment with Depo-Provera CI (ie, 4 intramuscular injections resulting in a cumulative dose of 600 mg) gained an average of 5.4 pounds (Depo-Provera CI US Prescribing Information 2016). In three large clinical trials using Depo-subQ 104, the mean weight gain was 3.5 pounds in the first year of use (Depo-subQ 104 US Prescribing Information 2016).

It is anticipated that change in weight in the proposed local tolerability study will be less than the weight gain associated with the chronic use of the approved 3-month contraceptives. Body weight will be measured at baseline and study exit.

1.3.2.5. Risk of Human Immunodeficiency Virus

Depo-Provera CI and Depo-subQ 104 provide no protection against sexually transmitted infections, including human immunodeficiency virus (HIV). While some observational data have suggested an association between the use of Depo-Provera CI and an increased risk of HIV acquisition (Morrison et al 2015, Ralph et al 2015, Polis et al 2016), the data are inconsistent and causality has not been established. In light of the inconclusive evidence, the World Health Organization (WHO) recently revised their MEC for contraceptive use (WHO 2017); per the revised criteria, women at low risk of HIV can continue using all hormonal methods of contraception including DMPA without restriction. Women who are at high risk of HIV, however, can use DMPA if they are informed of the possible increased risk of HIV acquisition among DMPA users, the uncertainty over a causal relationship, and how to minimize their risk of acquiring HIV. This study will not recruit women at high risk for HIV, but the information on how to minimize risk of acquiring HIV as well as the recommendation to use condoms for protection against HIV and sexually-transmitted infections will be provided to all subjects in this study.

1.3.2.6. Return to Fertility

Return to ovulation is delayed after stopping DMPA. Among 15 women who received multiple doses of Depo-subQ 104, the median time to ovulation was 10 months after the last injection; the earliest return to ovulation was 6 months after the last injection; and 12 women (80%) ovulated within 1 year of the last injection (Depo-subQ 104 US Prescribing Information 2016). Subcutaneous administration of DMPA is associated with a more delayed return to ovulation compared to the intramuscular route. In a comparative study, median time to return of ovulation after a single intramuscular injection of Depo-Provera CI (150 mg) and a single injection of

Depo-subQ (104 mg) were 183 and 212 days, respectively (although the difference was not statistically significant) (Jain et al 2004).

Return to fertility is also delayed. On the basis of the prescribing information, 68% of women who stop use may conceive within 12 months, 83% may conceive within 15 months, and 93% may conceive within 18 months from the last injection of Depo-Provera CI (Depo-Provera CI US Prescribing Information 2016). Among 28 women using Depo-subQ 104 for contraception who stopped treatment to become pregnant, only 1 became pregnant within a year of last injection and a second became pregnant 443 days after the last injection (7 women were lost to follow-up) (Depo-subQ 104 US Prescribing Information 2016).

In the local tolerability study, return to ovulation after the same-day administration of MPA in the total dose of 284 mg is likely to be delayed. Therefore, only women who are at low risk of pregnancy and who do not desire pregnancy for a minimum of 18 months will be enrolled into this study. Women will not be followed to assess return of ovulation.

1.3.2.7. Bone Mineral Density

In 2004, a boxed warning regarding skeletal health was added to the label of Depo-subQ 104 indicating that women who use these methods may lose significant bone mineral density (BMD) (Depo-subQ 104 US Prescribing Information 2016). The warning states that bone loss is greater with increasing duration of use and may not be completely reversible. It is unknown if use of Depo-subQ 104 during adolescence or early adulthood, a critical period of bone accretion, will reduce peak bone mass and increase the risk for osteoporotic fracture in later life. The label also states that Depo-subQ 104 should not be used as a long-term birth control method (ie, longer than 2 years) unless other birth control methods are considered inadequate. A similar boxed warning was added to the Depo-Provera CI prescribing information.

However, existing evidence indicates that the changes in BMD are largely reversible and comparable to the changes associated with the hypoestrogenism that occurs with pregnancy and lactation. Also, there is no evidence that long term use is actually associated with increased risk of fracture, the clinically relevant outcome (Kaunitz and Grimes 2011).

In the local tolerability study, any permanent adverse impact on BMD is unlikely. However, since the loss of BMD associated with the chronic use of Depo Provera CI and Depo-subQ 104 is of particular concern during adolescence and early adulthood, a critical period of bone accretion. Women younger than 18 years of age will not be eligible for this study.

1.3.2.8. Dose of MPA

The same-day administration of 120 mg/0.3 mL of TV-46046, 60 mg/0.3 mL of 1:1 saline diluted TV-46046, 0.3 mL of TV-46046 Placebo and 104 mg/0.65 mL of Depo-subQ 104 will result in a total dose of MPA of 284 mg; less than the 300 mg that was approved as a maximum dose of TV-46046 in Study TV-46046-WH-10075 and less than the dose of 300 mg used in an ongoing study sponsored by FHI 360 (Study #702179; IND 127097).

Several worldwide clinical studies have demonstrated that Depo-Provera CI at a dose of up to 450 mg, when administered by intramuscular injection, was safe and well-tolerated. Specifically, a clinical study in 61 women was conducted in Australia to determine the safety and efficacy of DMPA administered as 6-monthly intramuscular injections of 300 mg. Side effects were varied

but mild and generally related to changes in menstrual bleeding pattern (Mackay et al 1971). In the 4- and 5 ½-year-long clinical studies conducted in Thailand, 991 and 1132 women received Depo-Provera by intramuscular injection in doses of 300 and 400 mg every 6 months, respectively. In both studies, mild side effects included spotting, amenorrhea, and irregular menses (Schwallie and Assenzo 1972, McDaniel and Pardthaisong 1974). In a comparative clinical study in 1000 South African women, doses of 150 and 450 mg intramuscular injections of DMPA administered every 3 or 6 months, respectively, to 500 women each were well-tolerated with fewer women discontinuing the treatment in the 6-month group (Castle et al 1978). Depo-Provera is also currently approved in Canada as an intramuscular (im) injectable formulation at a dose of 50 mg weekly or 100 mg every 2 weeks intramuscularly for at least 6 months for the treatment of endometriosis (PRDepo-Provera and PRDepo-Provera-SC Product Monograph Canada 2013).

The safety of subcutaneous administration of a 300 mg dose of MPA has been demonstrated in an ongoing study (Study #702179; IND 127097) that compared the subcutaneous administration of 150 mg/mL (n=24) and 300 mg/mL (n=9) of Depo Provera CI with Depo-subQ 104 (n=9). Based on the interim analysis conducted when all enrolled subjects completed at least 7.5 months of follow-up, there were no serious adverse events and only two severe adverse events in the 300 mg group, both of which were considered not related to the product (an upper respiratory infection and thermal burn). As of April 2018, all nine women in the 300 mg group completed the study, and eight of them returned to ovulation within 18 months after the injection. Based on the interim data, the majority of participants in all groups had no clinically meaningful change from baseline in blood pressure or weight at Month 7.5. Possible suppression of adrenal function was evaluated by measuring serum cortisol levels throughout the study. There were no significant differences in cortisol levels observed in any of the three treatment groups.

In conclusion, the subcutaneous administration of MPA at the total dose of 284 mg proposed in the current study is expected to be safe. Vital signs, weight, adverse events and concomitant medications will be monitored during the study.

1.3.2.9. Other

According to the US Depo-subQ 104 and Depo-Provera CI labels, women with a strong family history of breast cancer should be carefully monitored when receiving DMPA. Five large case-control studies assessed the association between DMPA use and the risk of breast cancer: three studies suggested a slightly increased risk of breast cancer in the overall population of users (Lee et al 1987, WHO 1991, Shapiro et al 2000), and two studies demonstrated a statistically significant increased risk of breast cancer among recent DMPA users (Li et al 2012, Paul et al 1989). Women 40 years or older must have normal mammogram results in the last year prior to enrollment to be eligible for this study. If a woman 40 years or older has not had a mammogram in the past year, one will be scheduled between screening and enrollment.

Infrequent reports of anaphylaxis and anaphylactoid reaction have been associated with the use of Depo-Provera CI. On the day of the study, subjects will remain at the investigational center for observation for at least 1 hour after the last injection and receive counseling of possible signs of anaphylactic reaction prior to leaving the investigational center. The investigational centers will have an epinephrine injection on hand and easy access to emergency care in the unlikely event of an anaphylactic reaction.

Additional information regarding risks to human subjects related to TV-46046 may be found in the current IB.

Additional information regarding risks to subjects related to Depo-subQ 104 may be found in the Depo-subQ 104 Prescribing Information (Depo-subQ 104 US Prescribing Information 2016).

1.4. Study Design Rationale

1.4.1. General Study Design Rationale

Interim data from Study TV-46046-WH-10075 suggested that the subcutaneous administration of TV-46046 may be associated with a higher rate of ISRs than Depo-subQ 104. The lack of a direct comparison, the small sample size and the potential differences in ISR ascertainment complicate the interpretation of these results. The study described here will allow assessment and direct comparison of local tolerability between 120 mg/0.3 mL of TV-46046 and the reference drug, Depo-subQ 104. Inclusion of 60 mg/0.3mL of the 1:1 saline-diluted TV-46046 will enable investigation of a potential dose-dependent effect.

The proposed study is a single-group cross-over design in which 24 women will receive one of each of the four subcutaneous study injections in different quadrants of the abdomen approximately 1 hour apart (considered to be sufficient to eliminate carry-over effect of pain from previous injection). Each woman will serve as her own control to minimize the variability of treatment comparisons. Women will be randomized to one of 24 possible injection sequences (one subject per sequence) to counterbalance potential effects of injection order or abdominal quadrant on outcomes.

This study will recruit healthy subjects to eliminate confounding factors (comorbidities, comedications). Since TV-46046 is a hormonal contraceptive, only women will be enrolled into the study. Since the contraceptive dose and duration of TV-46046 action are unknown, enrollment will include women who are in menopause, or are premenopausal and are not pregnant, not wanting to become pregnant in the next 18 months, or at a low risk of pregnancy because they are sterilized, in an exclusively same-sex partnership, abstinent, in a monogamous relationship with a vasectomized partner, using non- hormonal IUD, or consistently using condoms.

In the ongoing TV46046-WH-10147 study, several cases of hypopigmentation had a later onset (ie, approximately 6 months post-injection) than observed in Study TV46046-WH-10075. In order to identify any late onset ISRs, subjects who have exited the study at Month 6 with no new or ongoing ISRs and who agree to return for a single follow-up visit between Months 9 and 12, will undergo ISR, adverse event, and concomitant medication evaluation, and vital sign and weight measurements at that time (see Section 3.4.5 for more detail).

1.4.2. Dosage Rationale

A 120 mg/0.3 mL dose of undiluted TV-46046 (containing 120 mg of MPA and 0.45 mg of docusate sodium sulfosuccinate [DSS]), will be used in this study. Based on interim data of the Phase 1 study (TV-46046-WH-10075), the PK/PD response of this dose appears consistent with the desired 6-month contraceptive protection and therefore may be similar to the dose used in future efficacy evaluations. The TV-46046 Placebo will be administered at a volume of 0.3 mL (containing 0.45 mg of DSS) to match the dose of DSS in the undiluted TV-46046 group. The

dose of 60 mg/0.3 mL of the 1:1 saline-diluted TV-46046 (containing 60 mg of MPA and 0.225 mg of DSS) was selected to evaluate if any treatment effect is potentially dose-dependent. Depo-subQ 104 will serve as an active control and will be administered at a dose of 104 mg/0.65 mL.

2. STUDY OBJECTIVES AND MEASURES/PARAMETERS

The purpose of this study is to evaluate the local tolerability associated with the subcutaneous administration of TV-46046, and inform next steps of the TV-46046 development program.

2.1. Study Objectives

2.1.1. Primary Objective

The primary objective is to evaluate and compare local tolerability associated with subcutaneous administration of 120 mg/0.3 mL of TV-46046, 60 mg/0.3 mL of 1:1 saline-diluted TV-46046, 0.3 mL of TV-46046 Placebo, and 104 mg/0.65 mL of Depo-subQ Provera 104® (medroxyprogesterone acetate injectable suspension, 104 mg/0.65 mL, hereafter referred to as Depo-subQ 104).

2.1.2. Secondary Objectives

The secondary objectives of the study are the following:

- To evaluate and compare injection site pain associated with subcutaneous administration of 120 mg/0.3 mL of TV-46046, 60 mg/0.3 mL of 1:1 saline-diluted TV-46046, 0.3 mL of TV-46046 Placebo, and 104 mg/0.65 mL of Depo-subQ 104
- To evaluate the overall safety of subcutaneous, same-day administration of 120 mg/0.3 mL of TV-46046, 60 mg/0.3 mL of 1:1 saline diluted TV-46046, 0.3 mL of TV-46046 Placebo, and 104 mg/0.65 mL of Depo-subQ 104

2.2. Study Endpoints

2.2.1. Primary Endpoint

The primary endpoint is ISR, excluding injection site pain, as assessed by self-reports and direct observation for each injection at least twice on the day of injection (Day 0: immediately after [ie, as soon as possible but no later than 10 minutes upon removing the needle] and 1 hour [±5 minutes] after the injection), at Days 1, 3, 7 and 14, at Months 3 and 6, and at additional visits, if indicated.

2.2.2. Secondary Endpoints

2.2.2.1. Injection Site Pain

- Injection site pain for each injection assessed by:
  - Numerical Rating Scale (NRS) score twice on the day of injection (Day 0: immediately [ie, as soon as possible but no later than 10 minutes upon removing the needle] after and 1 hour [±5 minutes] after the injection)
  - Self-reports using the NRS on Days 1, 3, 7 and 14, at Months 3 and 6, and at additional visits, if indicated

- Subjects' perception of injection site pain as assessed by an overall ranking of the 4 study injections from least [1] to most [4] painful on the day of injection (Day 0)

2.2.2.2. Overall Safety

- Occurrence of adverse events
- Use of concomitant medications
- Change in vital signs and weight

3. INVESTIGATIONAL PLAN

3.1. General Study Design

This is a randomized, crossover, single-center, Phase 1 study to evaluate and compare local tolerability following subcutaneous administration of TV-46046, diluted TV-46046, TV-46046 Placebo, and Depo-subQ 104, in 24 healthy female subjects 18 to 50 years of age. After signing the informed consent form, eligible subjects will be enrolled and receive one of each of the four subcutaneous study injections in each abdominal quadrant: 120 mg/0.3 mL of TV-46046, 60 mg/0.3 mL of 1:1 saline diluted TV-46046, 0.3 mL of TV-46046 Placebo and 104 mg/0.65 mL Depo-subQ 104 per the assigned sequence. Injections will be administered in different quadrants of the abdomen, each separated by approximately 1 hour (sufficient time to eliminate carry-over effect of pain at previous injection). Subjects will be randomized to one of 24 different injection sequences (one subject per sequence) to counterbalance potential effects of injection order or abdominal quadrant on study outcomes.

All subjects will be followed for at least 6 months after receiving their injections. Local tolerability will be assessed by evaluating ISRs at least twice on the day of the study injections (Day 0: immediately after [ie, as soon as possible but no later than 10 minutes upon removing the needle] and 1 hour [±5 minutes] after the injection), at Days 1, 3, 7, and 14; at Months 3 and 6; and at other visits, if indicated. Subjects with unresolved ISR(s) will be followed monthly through the resolution of ISR(s) or Month 18, whichever comes first.

Subjects will assess their injection site pain using an 11-point NRS (0 = no pain at all; 10 = worst pain) twice on the day of injection (Day 0: immediately after [ie, as soon as possible but no later than 10 minutes upon removing the needle] and 1 hour [±5 minutes] after the injection). In addition, 1 hour after the fourth (final) injection subjects will provide an overall ranking of administrations from least [1] to most [4] painful. Thereafter, injection site pain will be assessed by self-reports at all subsequent visits. Adverse events and concomitant medications will be recorded throughout the study. Vital signs and weight will be measured at baseline, Month 6, and study exit. The assessments and procedures performed during each study visit are detailed in Table 1 and Section 3.4.

3.2. Subject Eligibility

Prospective waivers (exceptions) from study eligibility criteria to allow subjects to enter a study are not granted by Teva (see Section 10.1.2).

3.2.1. Subject Inclusion Criteria

Subjects may be included in the study if they meet all of the following criteria:

a. has a low risk of pregnancy (ie, sterilized, in exclusively same-sex partnership, in menopause and/or post-menopausal, abstinent, in monogamous relationship with vasectomized partner, using non-hormonal intrauterine device (IUD), or consistent use of condoms)

b. is in good general health as determined by a medical history

c. is 18 to 50 years of age (inclusive)
d. is willing to provide informed consent and follow all study requirements
e. is not pregnant and does not have desire to become pregnant in the subsequent 18 months
f. had a normal mammogram within the last year, if 40 years or older
g. has no skin disorders or skin allergies

3.2.2. Subject Exclusion Criteria

Subjects will be excluded from participating in this study if they meet any of the following criteria:

a. has hypertension,
   - systolic blood pressure (BP) ≥160 mm Hg or diastolic BP ≥100 mm Hg or
   - vascular disease
b. has ischemic heart disease or a history of ischemic heart disease
c. has a history of stroke
d. has a history of thromboembolic event(s)
e. has systemic lupus erythematosus or
   - positive (or unknown) antiphospholipid antibodies or
   - severe thrombocytopenia
f. has rheumatoid arthritis and is undergoing immunosuppressive therapy
g. has migraine with aura
h. has unexplained vaginal bleeding
i. has diabetes
j. has a strong family history of breast cancer (defined as one or more first degree relatives, breast cancer occurring before menopause in three or more family members, regardless of degree of relationship, or any male family member with breast cancer), or current or history of breast cancer, or undiagnosed mass detected by breast exam
k. has cervical cancer or a history of cervical cancer
l. has severe cirrhosis (decompensated) or liver tumors
m. has known significant renal disease
n. has a history of diagnosed clinical depression or bipolar disorder, with or without suicidal ideation, and/or history of suicide attempt
o. has, in the last two years, a history of either hospitalization or medication management for a psychiatric disorder that in the opinion of the investigator would

make study participation unsafe, would interfere with adherence to study requirements or complicate data interpretation

p. is currently using hormonal contraception

q. had an injection of DMPA (Depo-Provera CI or Depo-subQ 104) in the past 12 months; or combined injectable in the last 3 months

r. has a known sensitivity to MPA or any inactive ingredients

s. is chronically using pain medication

t. has a plan to move to another location in the next 18 months

u. has, in the opinion of the investigator, a potentially elevated risk of HIV infection (eg, HIV-positive partner, IV drug use by self or by partner)

v. has any condition (social or medical), which in the opinion of the investigator would make study participation unsafe, would interfere with adherence to the clinical study requirements or would complicate data interpretation

3.3. Duration of Subject Participation

Screening for eligibility, enrollment, randomization and administration of study treatments can occur on the same day or may be split between two visits for logistical reasons. This will be followed by a treatment period of 6 months. If subjects have unresolved ISR(s) at Month 6, they will be followed until ISR resolution or Month 18, whichever comes first. Therefore, the minimal duration of subject participation is 6 months and the maximum is 18 months after the study treatment. See Section 11.5 for the definition of the end of the study.

3.4. Study Procedures

Before implementation, the protocol and all relevant study documents including recruiting materials will be approved by the investigational center's local Institutional Review Board (IRB)/Independent Ethics Committee (IEC) and by FHI 360's Protection of Human Subjects Committee (PHSC).

Description and timing of study procedures and assessments are summarized in Table 1. Detailed visit-specific information is provided in this section. Detailed descriptions of each assessment are provided in Section 5.1 and Section 5.2.

Table 1: Study Procedures and Assessments

Procedures and Assessments	Screening	Treatment Day					Scheduled Follow-Up Period			Additional Follow-Up Period, if Indicated
	Day -1	Day 0	Injection 1	Injection 2	Injection 3	Injection 4	Days 1, 3, 7, 14	Months 1, 2, 4, 5	Months 3 and 6[a]	Months 7, 8, 9, 10, 11, 12, 13, 14, 15, 16, 17, 18[b]
Informed consent	X									
Medical history	X									
Contraceptive history	X									
Inclusion and exclusion criteria	X	X								
Urine pregnancy test		X					[X]	[X]	[X]	[X]
Blood pressure	X									
Vital Signs (Blood pressure, pulse, respiration rate, temperature)[c]		X							X	[X]
Weight [c]		X							X	[X]
Mammogram [d]	[X]									
Breast exam	X									
Randomization		X								
IMP injection			X	X	X	X				
Injection site pain NRS assessment [e]			X	X	X	X	X		[X]	[X]
ISR evaluation [f]			X	X	X	X	X	[X]	X	X

Procedures and Assessments	Screening	Treatment Day					Scheduled Follow-Up Period			Additional Follow-Up Period, if Indicated
	Day -1	Day 0	Injection 1	Injection 2	Injection 3	Injection 4	Days 1, 3, 7, 14	Months 1, 2, 4, 5	Months 3 and 6[a]	Months 7, 8, 9, 10, 11, 12, 13, 14, 15, 16, 17, 18[b]
Adverse event assessment [f,g]		[X]	[X]	[X]	[X]	[X]	[X]	X	X	[X]
Concomitant medication assessment [g]		X					[X]	X	X	[X]

[a] If the subject does not have any ISRs at Month 6 and subject does not agree to return for an additional post-6 month follow up visit, Month 6 will be her final visit. In addition to ISR, adverse event, and concomitant medication evaluation, her weight and vital signs will be measured at that time. Subjects who complete the study at Month 6 will be instructed to contact the clinic if any ISRs develop after their final visit. If the subject is reported to have ISR(s) at Month 6, she will be instructed to return to the clinic monthly until ISR resolution or Month 18, whichever is earlier. In this case, the Month 6 procedures will be repeated at her final visit.

[b] Subjects who have exited the study with no new or ongoing ISRs at Month 6 and who agree (providing written informed consent) to return for a single follow-up visit between Months 9 and 12, will undergo ISR, adverse event, and concomitant medication evaluation, and vital sign and weight measurements at that time. If the subject does not have any ISRs at this visit, it will be her final visit and the subject will be instructed to contact the clinic if any ISRs develop after their final visit. If the subject does have ISR(s) at this visit, she will be instructed to return to the clinic monthly until the ISR resolution or Month 18, whichever is earlier. In this case, ISR, adverse event, and concomitant medication evaluation, and vital sign and weight measurements will be repeated at her final visit.

[c] Vital signs and weight will be measured on Enrollment/Injection Day (Day 0) and Month 6, and at study exit.

[d] Normal mammogram within 1 year prior to enrollment, if 40 years or older.

[e] For each injection, injection site pain will be evaluated twice on the day of the study drug injection (Day 0: immediately after [ie, as soon as possible but no later than 10 minutes upon removing the needle] and 1 hour (±5 minutes) after the injection using NRS. For all subsequent visits, NRS will be used only if injection site pain is self-reported. One hour after the fourth (final) injection subjects will provide an overall ranking of all four administrations from least [1] to most [4] painful.

[f] For each injection, ISR will be evaluated at least twice on the day of the study drug injection (Day 0: immediately after [ie, as soon as possible but no later than 10 minutes upon removing the needle] and 1 hour (±5 minutes) after the injection, and on Days 1, 3, 7, and 14, Months 3 and 6, and at additional visits if indicated. Each injection site will be marked at the time of the injection and a photo taken for reference after the fourth injection.

[g] Study staff will inquire about adverse events and concomitant medications at Months 1, 2, 3, 4, 5 and 6 and at study exit, but will document adverse events and concomitant medications at any visit where the subject reports any new or changed adverse events or concomitant medications. For subjects who have previously exited the study at Month 6 and who agree to return for a single follow-up visit between Months 9 and 12, study staff will inquire about adverse events and concomitant medications during this visit and at study exit.

IMP=investigational medicinal product; NRS=Numerical Rating Scale; ISR=injection site reaction
NOTE: X=required, [X]=if indicated

3.4.1. Procedures for Screening Visit

Women may be recruited through various channels, including via regular informational sessions held at the study clinic where patients are informed about potential research opportunities. Women who express interest to participate will undergo an informed consent process administered by trained staff members including information about the study objectives, design and procedures, and potential risks and benefits of participation. A signed and dated informed consent form (ICF) will be obtained before screening procedures commence (see Section 11.3). After informed consent is obtained, the subject will be assigned a subject identification number in successive order of inclusion. Demographic information will be collected, a breast exam and blood pressure measurement will be performed and other eligibility criteria verified including medical and contraceptive history. The investigational center may conduct clinical procedures per investigational center-specific standard of care that are not required per the study protocol (eg, pelvic exam).

If the participant has not had a mammogram in the past year and is 40 years or older, one will be scheduled prior to enrollment. In consultation with FHI 360, a subject who is screened and does not meet eligibility criteria may be considered for re-screening if the non-met criteria change

3.4.2. Procedures for Enrollment/Injections Visit

On the day of enrollment, a urine pregnancy test will be performed; weight and vital signs will be measured (ie, blood pressure, pulse rate, respiration rate and temperature). Mammogram results from the prior year will be verified for women 40 years and older. Subject eligibility will be confirmed and enrollment/randomization procedures will be performed.

After eligibility is confirmed, the subject will be enrolled into the study and any ongoing medication will be documented as a concomitant medication. The enrolled subject will be randomized to a treatment sequence. An unblinded study staff member will enter subject information into OpenClinica's RANDOMISE module, which will display the randomly selected treatment sequence. Date and time of randomization will be recorded on the study source documentation and case report form (CRF). All unblinded staff involved in randomization procedures will conceal the study drug sequence assignment and all records containing allocation sequence from the subject and the blinded staff (See Section 4.3 on Blinding/Unblinding for more detail).

The subject will receive one of each of the four injections per the assigned sequence separated by approximately 1 hour: 120 mg/0.3 mL of TV-46046 injected with 23G needle, 60 mg/0.3 mL of 1:1 saline-diluted TV-46046 injected with 23G needle, 0.3 mL of TV-46046 Placebo injected with 23G needle, and 104 mg/0.65 mL of Depo-subQ 104 injected with 26G needle. Shielding the needle and syringe from the view of the subject, an unblinded study staff member qualified and experienced in administering parenteral drugs, will administer all four injections subcutaneously in four abdominal quadrants starting with the subject's right upper quadrant (injection A) and progressing in a clockwise manner to the left upper quadrant (injection B), left lower quadrant (injection C), and right lower quadrant (injection D). Each injection site will be marked at the time of the injection and a photo taken for reference after the fourth injection. For each injection, ISRs and injection site pain will be evaluated immediately after (ie, as soon as possible but no later than 10 minutes upon removing the needle) and 1 hour (±5 minutes) after

each injection (right before the next injection). In addition, 1 hour after the fourth (final) injection subjects will provide an overall ranking of administrations from least [1] to most [4] painful. See Section 5.1.1 for more information on assessment of ISRs and injection site pain. In addition, detailed instructions for NRS assessment and documentation will be provided in the Study Manual.

In summary, the following procedures will be conducted on the Enrollment/Injection Visit (Day 0):

- eligibility confirmed
- urine pregnancy test performed
- vital signs and weight measured
- adverse events documented if occurring after ICF is signed
- concomitant medications recorded
- treatment sequence allocation obtained
- study drug injections administered per treatment sequence allocation
- injection sites evaluated for ISRs
- injection site pain assessed by NRS
- all four injections ranked from least [1] to most [4] painful
- photo of the four marked injection sites taken

3.4.3. Follow-up/Final Visits

All subjects will return to the clinic on Day 1, Day 3, Day 7, Day 14, Month 1 and then monthly through Month 6. During Days 1, 3, 7, and 14 and Months 3 and 6 visits, ISRs and injection site pain for each of the four study injections will be evaluated following procedures outlined in Section 5.1. In addition, at monthly visits between Months 1 and 6, information on adverse event and concomitant medications will be collected. If the subject does not have any ISRs at Month 6, Month 6 will be her final visit, unless she agrees to return for additional follow up as detailed in Section 3.4.5. In addition to ISR, adverse event, and concomitant medication evaluation, her weight and vital signs will be measured at that time. Subjects who complete the study at Month 6 will be instructed to contact the clinic if any ISRs develop after their final visit. If any ISRs are identified or still ongoing at Month 6, monthly follow-up visits for ISR evaluation will be scheduled until the ISR is resolved or Month 18, whichever is earlier. In this case, the Month 6 procedures will be repeated at her final visit.

The following procedures will be performed during Follow-up/Final visits:

- injection sites evaluated for ongoing and/or new ISRs (Days 1, 3, 7, and 14 and Months 3 and 6 and at study exit)
- injection site pain assessed if self-reported (Days 1, 3, 7, and 14 and Months 3 and 6, and other visits if appropriate)

- adverse event and concomitant medication inquiry conducted (at Months 1, 2, 3, 4, 5 and 6 and at study exit)
- vital signs and weight measured (Month 6 and at study exit)

3.4.4. Unscheduled Visits

An unscheduled visit may be performed at any time during the study at the subject's request or as deemed necessary by the investigator. The date and reason for the unscheduled visit will be recorded on the CRF as well as any other data obtained, if applicable (eg, ISRs, adverse events, concomitant medications and treatments, and results from procedures or tests).

3.4.5. Post-6 Month Follow-up

In order to identify any late onset ISRs, subjects who have exited the study with no new or ongoing ISRs at Month 6 and who agree (providing written informed consent) to return for a single follow-up visit between Months 9 and 12, will undergo ISR, adverse event and concomitant medication evaluation, and vital sign and weight measurements at that time. If any ISRs are identified or still ongoing at this additional visit between Months 9 and 12, monthly follow-up visits for ISR evaluation will be scheduled until the ISR is resolved or Month 18, whichever is earlier. In this case, ISR, adverse event, and concomitant medication evaluation, and vital sign and weight measurements will be repeated at her final visit. If the subject reports injection site pain at any of these visits, she will be asked to use the NRS to evaluate the intensity of pain in order to determine if it meets the definition of an adverse event. If the subject does not have any ISRs at this visit, it will be her final visit. However, the subject will be instructed to return to the clinic if any ISRs develop after her final visit. In that case, she will be consented, her status will change from completed the study to active and she will be followed until ISR resolution or Month 18, whichever is earlier, as described above.

4. TREATMENT OF SUBJECTS

4.1. Investigational Medicinal Products Administered During the Study

The IMPs used in this study will be supplied by the sponsor and are described in Table 2.

4.1.1. Test Investigational Medicinal Product

Medroxyprogesterone acetate injectable suspension 400 mg/mL (TV-46046) will be provided by the sponsor as a sterile suspension. TV-46046 will be used at 2 different concentrations: 0.3 mL of 400 mg/mL (undiluted) and 0.3 mL of 200 mg/mL (saline diluted). To obtain the 200 mg/mL concentration, TV-46046 will be diluted with sterile saline by the delegated study staff following the sponsor's recommendations. Saline will be provided to the site by either the contract research organization or the sponsor. Dilution procedures will be followed per the Pharmacy Manual.

Each TV-46046 dose will be presented in a 3 mL United States Pharmacopeia (USP) Type 1 clear glass vial, with a 13-mm rubber stopper, and a 13-mm aluminum seal covered with a green cap (STERI TAMP® single-use tamper evident sticker). The test IMP will be administered with a 23 gauge, 3/8" safety needle.

4.1.2. Reference Investigational Medicinal Product

Depo-subQ 104 is the reference IMP. It is indicated for prevention of pregnancy at a dose of 104 mg administered every 3 months by a 0.65 mL subcutaneous injection. For this study, Depo-subQ will be supplied as a single-unit dose of 104 mg/0.65 mL prefilled glass syringes pre-packaged with 26 gauge 3/8" needle. Detailed composition and injection instructions are contained in the Depo-subQ 104 Prescribing Information (Depo-subQ 104 US Prescribing Information 2016).

4.1.3. Placebo Investigational Medicinal Product

Medroxyprogesterone acetate injectable suspension 400 mg/mL (TV-46046), Placebo (or TV-46046 Placebo) will be supplied in a 2 mL clear glass vial (filling volume 1.2 mL), with a rubber stopper and covered with a white cap.

Similar to TV-46046, 0.3 mL of TV-46046 Placebo will be administered with a 23 gauge, 3/8" safety needle.

4.2. Treatment of Subjects

Subjects will receive all four study injections (120 mg/0.3 mL of TV-46046, 60 mg/0.3 mL of 1:1 saline-diluted TV-46046, 0.3 mL of TV-46046 Placebo and 104 mg/0.65 mL of Depo subQ 104) on the same day approximately 1 hour apart. The four injections will be administered subcutaneously in four abdominal quadrants starting with the subject's right upper quadrant (injection A) and progressing in a clockwise manner to the left upper quadrant (injection B), left lower quadrant (injection C), and right lower quadrant (injection D), per the assigned sequence. The study staff administering the injections will be instructed not to apply bandages, and to avoid touching or massaging the injection site. In case of bleeding, a gentle application of the cotton ball for several minutes will be recommended (ie, no application of ice or bandages). Subjects

will remain at the investigational center for at least 1 hour after the last injection. The investigational center will have an epinephrine injection available and easy access to emergency care in the unlikely event of anaphylaxis or anaphylactoid reaction.

Table 2: Investigational Medicinal Products Used in the Study

IMP name	TV-46046		TV-46046 Placebo		Reference IMP	
Trade name and INN, if applicable, or company assigned number	Medroxyprogesterone acetate		TV-46046 Placebo		Medroxyprogesterone acetate injectable suspension	
Formulation	Medroxy-progesterone Acetate	400 mg/mL	NA	NA	Medroxy-progesterone acetate	104 mg
	Docusate sodium	1.5 mg/mL	Docusate sodium	1.5 mg/mL	Methylparaben	1.040 mg
	Polyethylene glycol 3350	20.3 mg/mL	Polyethylene glycol 3350	20.3 mg/mL	Propylparaben	0.098 mg
	Sodium sulfate anh.	11 mg/mL	Sodium sulfate anh.	11 mg/mL	Sodium Chloride	5.200 mg
	Monobasic sodium phosphate anh.	0.25 mg/mL	Monobasic sodium phosphate anh.	0.25 mg/mL	Polyethylene Glycol	18.688 mg
	Dibasic Sodium phosphate anh.	0.52 mg/mL	Dibasic Sodium phosphate anh.	0.52 mg/mL	Polysorbate 80	1.950 mg
	L-Methionine	1.5 mg/mL	L-Methionine	1.5 mg/mL	Monobasic Sodium Phosphate · H_2O	0.451 mg
	Water for Injection	qs to 1 mL	Water for Injection	qs to 1 mL	Dibasic Sodium Phosphate · $12H_2O$	0.382 mg
	NA	NA	NA	NA	Methionine	0.975 mg
	NA	NA	NA	NA	Povidone	3.250 mg
	NA	NA	NA	NA	Water for injection	qs
Unit dose strength(s)/ Dosage level(s)	120 mg/0.3 mL<br>(diluted TV-46046: 60 mg/0.3 mL)		0.3 mL		104 mg/0.65 mL	
Route of administration	SC injection<br>(Sol Care Safety syringe 0.5 ml,<br>with fixed needle 23G x 3/8”<br>[0.6mm x 10mm]		SC injection<br>(Sol Care Safety syringe 0.5 ml,<br>with fixed needle 23G x 3/8”<br>[0.6mm x 10mm]		SC injection with 26G needle	

IMP=investigational medicinal product; INN=international proprietary name; NA=not applicable; anh=anhydrous; qs=quantify sufficient; SC=subcutaneous; G=gauge.

4.3. Blinding/Unblinding

The study will be partially blinded due to differences in appearance and volume of the treatments. Designated unblinded study staff will conduct randomization procedures. The staff preparing and administering injections will also be unblinded to treatment sequence but will be trained to shield the syringe prior to and at the time of injection from view of the subject and study staff assessing ISRs. Additional study staff will be unblinded to provide quality assurance and oversight of injection preparation and administration. These individuals will not be involved in any additional conduct of study procedures, collection of ISR data, or assessment of any adverse events.

Study subjects and staff conducting follow-up interviews, and recording of complaints, symptoms and clinical findings, will be unaware of sequence assignments throughout the study. Specifically, blinded study staff will be responsible for evaluating ISRs, adverse events, concomitant medication use, and measuring vital signs and weight at all appropriate study timepoints. Injection site pain will be assessed in a blinded manner by subjects themselves.

Records of treatment sequence assignment will be maintained in a secure manner accessible only to unblinded study staff. FHI 360 will assign a blinded clinical monitor to conduct routine investigational center monitoring visits and a separate unblinded clinical monitor to reconcile study supplies dispensed and maintained at the investigational center, and review randomization and dosing documentation during the study. FHI 360 and sponsor project team members involved in assessment of adverse events, data analysis, or results interpretation will be blinded to treatment sequence until at least the first interim analysis, at which time results may be reviewed by treatment to inform decisions whether to stop enrollment or modify the study.

4.3.1. Unblinding During the Study

Because of the cross-over design of one day treatment only and long-term follow-up/observation nature of the study drugs, emergency unblinding for medical reasons will be of limited (if any) value, and therefore no prior plans for emergency unblinding will be put in place.

For adverse events that are defined as a Suspected Unexpected Serious Adverse Reaction (SUSAR) (ie, reasonable possibility that adverse event is related to treatment; Section 5.2.5), Global Patient Safety and Pharmacovigilance (GPSP) will request that the treatment code be revealed to comply with regulatory requirements. The report will be provided in an unblinded manner for regulatory submission. If this occurs, blinding will be maintained for the investigator and for other personnel involved in the conduct, analysis, and reporting of the data.

All cases of inadvertent unblinding will be handled per FHI 360's SOPs.

4.4. Stopping Rules and Discontinuation Criteria

The investigational center will be advised to pause enrollment if at any time two or more ISRs graded as severe adverse events, two cases of injection site discoloration (hypopigmentation) ongoing for more than 7 days, or two serious and related adverse events are reported in the study across all injection sites. Such occurrences will trigger an unplanned review of interim data by a Study Review Committee (SRC), at which time a recommendation to halt the study or to reinitiate enrollment may be made. Other planned reviews will take place as scheduled (see

Section 4.5), at which time the SRC could advise that the study stop or be modified based on concerns for safety of subjects or definitive conclusions.

Since most of the study drugs are expected to remain in the body months after injection, study discontinuation will not be the same as treatment discontinuation. Study staff will explain to subjects who wish to discontinue the study early that it is important to stay in the study until safety assessments are completed. However, a subject may discontinue participation in the study at any time for any reason (eg, withdrawal of consent or an adverse event) without loss of other benefits or services to which they may be entitled. All subjects who discontinue early will be followed according to Subject Withdrawal Criteria and Procedures (Section 4.6.1). Reasons for discontinuation from the study will be recorded on the appropriate CRF.

Others reasons the study may be stopped include:

- FHI 360's PHSC recommends terminating the study
- Local IRB(s)/IEC(s) recommend terminating the study
- The FDA requests that the study be discontinued or placed on hold.
- The sponsor decides to reduce the scope (eg, reduce sample size, drop one or more study groups) or terminate the study. The sponsor may terminate the study at any time for any reason.

If the whole study is stopped, the subjects that are terminated early will be followed according to Subject Withdrawal Criteria and Procedures (Section 4.6.1).

4.5. Interim Data Reviews

The study will be overseen by an internal SRC, the composition and responsibilities of which will be detailed in a separate SRC Operational Plan. In addition to unplanned reviews that may be triggered by the occurrence of ISRs, serious and related adverse events, and/or injection site discoloration (hypopigmentation) events, the SRC may convene for a single planned review of interim data when approximately 50% of study subjects have been enrolled and treated. If, however, enrollment is predicted to take less than 2 months, then the interim review may instead be scheduled to occur after all subjects have been treated. At the time of any planned or unplanned interim review of data, the SRC may recommend that the trial be modified or halted to ensure the safety and well-being of study subjects. There will be no adjustment to type I error to account for any interim reviews of study data.

4.6. Prior and Concomitant Therapy or Medication

Any medication a subject uses between the enrollment and final visit will be recorded as a concomitant on the appropriate CRF. During scheduled Months 1, 2, 3, 4, 5 and 6 visits the investigator will ask subjects whether they have taken any medications, including over-the-counter medications, vitamins, or herbal or nutritional supplements, since the previous visit. During all other scheduled and unscheduled visits information on the use of concomitant medications will be documented only if reported by the subject. Generic or trade name, indication, route of IMP administration, dosage, and start and end dates will be recorded.

Subjects who are taking pain medications on a chronic basis will not be eligible for the study. There will be no prohibited medications during the study; however, application of bandages to injection sites will be discouraged.

4.6.1. Subject Withdrawal Criteria and Procedures

In accordance with the Declaration of Helsinki, each subject is free to withdraw from the study at any time. The investigator also has the right to withdraw a subject from the study in the event of intercurrent illness, adverse events, pregnancy (see Section 5) or other reasons concerning the health or well-being of the subject, or in the event of lack of cooperation.

Should a subject decide to withdraw from the study, or should the investigator decide to withdraw the subject, every reasonable effort will be made to assess information relevant to the endpoints at the time of discontinuation. In this study this mainly includes assessment of ISRs. The final visit procedures will be followed for all subjects who withdraw, if possible (see Section 3.4.3).

The reason for and date of withdrawal from the study must be recorded on the source documentation and transcribed onto the CRF. If a subject withdraws consent, every attempt will be made to determine the reason.

If the investigator determines that an adverse event is related to the test IMP, monitoring will continue until the adverse event has resolved or stabilized, the subject exited the study or the subject has reached the end of the follow-up period. The investigator must inform FHI 360 as soon as possible of all subjects who are being considered for withdrawal due to adverse events. Additional reports must be provided when requested.

The study product remains in the body months after the injections, therefore, prompt withdrawal from the study treatment is not possible, and discontinuation from the study will not be the same as withdrawal from treatment. Study staff will explain to subjects who wish to discontinue from the study early that it is impossible to discontinue the treatment and that it is important to stay in the study until safety assessments are completed.

4.7. Procedures for Monitoring Subject Compliance

All IMPs will be injected by the investigator or other clinical personnel at the investigational center; therefore, monitoring of subject compliance will not be necessary.

4.8. Withdrawal of Subjects

4.8.1. Lost to Follow-up

If a subject fails to appear for a scheduled visit, at least 3 attempts to contact the subject will be made. If she does not return to clinic while the study is ongoing then she will be considered “presumed lost to follow-up”, but her file will remain open until study closeout. If the subject does not return before the study is closed, she will be classified as lost to follow-up. The lost to follow-up designation will not be made for any subject until the closing date of the study.

5. SAFETY AND TOLERABIILITY MEASUREMENTS AND ASSESSMENTS

5.1. Safety and Tolerability Measurements

5.1.1. Local Tolerability

The four study injections will be administered in different quadrants of the abdomen, each separated by approximately 1 hour (sufficient time to eliminate carry-over effect of pain at previous injection). The injections will be administered subcutaneously in four abdominal quadrants starting with the subject's right upper quadrant (injection A) and progressing in a clockwise manner to the left upper quadrant (injection B), left lower quadrant (injection C), and right lower quadrant (injection D), per the randomized sequence. Each injection site will be marked at the time of the injection and a photo will be taken of all four injection sites for future identification.

In this study local tolerability will be assessed by occurrence of ISRs including but not limited to erythema (redness), swelling, pruritus (itching), bleeding, bruising, injection site discoloration (eg, hypopigmentation), or atrophy (ie, dimple). Injection site pain will be evaluated and analyzed as a separate study endpoint (see Section 5.1.2).

The site of injection will be evaluated for possible ISRs after each of the four injections at least twice during the enrollment/injection visit (Day 0) immediately after (ie, as soon as possible but no later than 10 minutes upon removing the needle) and 1 hour (±5 minutes) after each injection (right before the next injection). Given the study design and assessment schedule, injection sites for the first, second and third injections may be evaluated more than twice. Thereafter, injection sites will be monitored for the progress of ongoing and/or occurrence of new ISRs at Days 1, 3, 7, and 14; at Months 3 and 6 and at other visits, if indicated. Subjects with no new or ongoing ISRs at their final visit will complete the study but be instructed to contact the clinic if new ISRs develop after their final visit (see Section 3.4.3 and Section 3.4.5). Subjects with ISRs at their final visit will be followed monthly until ISR resolution or Month 18, whichever comes first.

ISRs will be evaluated at all study visits by subject's self-reports and visual examination of the site of injection by blinded study staff. The study staff will be instructed to use non-leading questions (eg, how does the injection site feel, do you have any new complaints about the injection site since last visit) for ISR ascertainment. All findings will be recorded for each of the four study injections on the appropriate study CRFs and clinical notes. Photos of the injection site may be taken to supplement documentation of ISRs any time during the study. If necessary, a consultation with an appropriate clinical expert may be scheduled. All cases of injection site discoloration (hypopigmentation) will be photographed and consulted by a dermatologist.

5.1.2. Injection Site Pain

For the purpose of this protocol, injection site pain will include pain associated with insertion of the needle, injecting of the drug, and any pain or tenderness at/around the injection site. On the day of injection (Day 0) injection site pain will be evaluated twice: immediately after (ie, as soon as possible but no later than 10 minutes upon removing the needle) and 1 hour (±5 minutes) after

each injection (right before the next injection). Subjects will assess their injection site pain using an 11-point NRS by indicating a number between 0 (no pain) and 10 (worst pain). In addition, 1 hour after the fourth (final) injection subjects will provide an overall ranking of all four administrations from least [1] to most [4] painful. Thereafter, injection site pain will be evaluated by self-reports on Days 1, 3, 7, and 14, Month 3 and 6, and at additional visits, if appropriate. If the subject reports injection site pain at any of these visits, she will be asked to use the NRS to evaluate the intensity of pain in order to determine if it meets the definition of an adverse event.

5.2. Safety Assessments

The following safety and tolerability measures will be implemented throughout the study at the time points listed in Table 1.

- vital signs and weight will be measured at Day 0 and Month 6 and study exit
- adverse events and concomitant medications will be evaluated at Day 0, at Months 1 through 6, and at study exit
- information about adverse events and concomitant medications will not be actively solicited but documented if reported on Days 1, 3, 7, and 14 and unscheduled visits

5.2.1. Definition of an Adverse Event

An adverse event is any untoward medical occurrence in a subject administered a pharmaceutical product, regardless of whether it has a causal relationship with the treatment.

In this study, any adverse event occurring after the subject has signed the ICF through the end of the follow up period will be recorded and reported as an adverse event.

An adverse event can, therefore, be any unfavorable and unintended physical sign, symptom, or laboratory parameter that develops or worsens in severity during the course of this study, or significant worsening of any concurrent disease, whether or not considered related to TV-46046, diluted TV-46046, TV-46046 Placebo or Depo-subQ 104. A new condition or the worsening of a pre-existing condition will be considered an adverse event. Stable chronic conditions (such as arthritis) that are present before study entry and do not worsen during this study will not be considered adverse events.

Accordingly, an adverse event can include any of the following:

- intercurrent illnesses
- physical injuries
- events possibly related to concomitant medication
- significant worsening (change in nature, severity, or frequency) of pre-existing conditions (Note: A condition recorded as pre-existing that is intermittently symptomatic [eg, headache] should be recorded as an adverse event if frequency of its occurrence increases during this study.)
- drug interactions
- laboratory or diagnostic test abnormalities, that result in the withdrawal of the subject from the study, are associated with clinical signs and symptoms or a serious adverse

event, or require medical treatment or further diagnostic work-up, or are considered by the investigator to be clinically significant (Note: Abnormal laboratory test results at the screening visit that preclude a subject from entering the study or receiving study treatment are not considered adverse events.)

- Any physical examination, vital signs measurement, or other safety assessment finding that is judged by the investigator as a clinically significant change (worsening) compared with a baseline value will be considered an adverse event. For the purpose of this study, the following will be considered adverse events:
  - Irregular vaginal bleeding that requires medical intervention or meets the definition of "serious adverse event" (See Section 5.2.5)
  - Injection site pain with a NRS score of 7 to 10
  - Injection site pruritus/itching if itching localized to the injection site requiring ≥48 hours treatment OR generalized itching causing inability to perform usual social and functional activities
  - Injection site erythema/redness of ≥5 cm in diameter (or ≥ 25 cm^2 surface area) or greater than minimal interference with usual social and functional activities
  - Injection site induration/swelling of ≥5 cm in diameter (or ≥ 25 cm^2 surface area) or greater than minimal interference with usual social and functional activities
  - Any ISR that meets the definition of "serious adverse event"
  - Pre-existing diseases or conditions that worsen during the study.

5.2.2. Recording and Reporting Adverse Events

For adverse event recording, the study period is defined for each subject as that time period from signature of the ICF through the end of the subject's follow-up period.

All adverse events that occur during the defined study period must be recorded on the source documentation and transcribed onto the CRF, regardless of the severity of the event or judged relationship to the IMP. For serious adverse events, the Serious and Protocol Defined Adverse Event Form must also be completed and the event must be reported immediately (See Section 5.2.5.3). The investigator does not need to actively monitor subjects for adverse events once their follow-up period has ended. Serious adverse events occurring in a subject after study discontinuation will be reported to the sponsor if the investigator becomes aware of them, following the procedures described in Section 5.2.5.3.

At the Day 0, Months 1 through 6 and study exit, the investigator must question the subject about adverse events by asking an open-ended question such as, "Have you had any unusual symptoms or medical problems since the last visit? If yes, please describe." At the scheduled Day 1, 3, 7, and 14 and unscheduled visits the information about adverse events will not be actively solicited but documented if spontaneously reported by the subject. All observed or reported signs and symptoms will be recorded individually, except when considered manifestations of a medical condition or disease state. A precise diagnosis will be recorded whenever possible. When such a diagnosis is made, all related signs, symptoms, and any test findings will be recorded collectively

as a single diagnosis on the CRF and, if it is a serious adverse event, on the Serious Adverse Event Form.

The clinical course of each adverse event will be monitored at suitable intervals until resolved or stabilized or returned to baseline, or until a determination of a cause unrelated to the treatment or study procedure is made.

The onset and end dates and times, action taken regarding IMP, treatment administered, and outcome for each adverse event must be recorded on the source documentation and transcribed onto the CRF.

The relationship of each adverse event to IMP and study procedures, and the severity and seriousness of each adverse event, as judged by the investigator, must be recorded as described below.

5.2.3. Severity of an Adverse Event

The severity of adverse events including ISRs must be recorded as one of the following:

Mild: No limitation of usual activities

Moderate: Some limitation of usual activities

Severe: Inability to carry out usual activities

5.2.4. Relationship of an Adverse Event to the IMP

The relationship of an adverse event to the IMP will be characterized per the table below where possible, noting it may not be possible to assess relatedness of systemic adverse events due to the cross-over nature of the study.

Term	Definition	Clarification
No reasonable possibility (not related)	This category applies to adverse events that, after careful consideration, are clearly due to extraneous causes (disease, environment, etc.) or to adverse events that, after careful medical consideration at the time they are evaluated, are judged to be unrelated to the IMP.	The relationship of an adverse event may be considered “no reasonable possibility” if it is clearly due to extraneous causes or if at least 2 of the following apply:<br>• It does not follow a reasonable temporal sequence from the administration of the IMP.<br>• It could readily have been produced by the subject’s clinical state, environmental, or toxic factors, or other modes of therapy administered to the subject.<br>• It does not follow a known pattern of response to the IMP.<br>• It does not reappear or worsen when the IMP is readministered.

Term	Definition	Clarification
Reasonable possibility (related)	This category applies to adverse events for which, after careful medical consideration at the time they are evaluated, a connection with the IMP administration cannot be ruled out with certainty.	The relationship of an adverse event may be considered “reasonable possibility” if at least 2 of the following apply:<br>• It follows a reasonable temporal sequence from administration of the IMP.<br>• It cannot be reasonably explained by the known characteristics of the subject’s clinical state, environmental or toxic factors or other modes of therapy administered to the subject.<br>• It disappears or decreases on cessation or reduction in dose. There are important exceptions when an adverse event does not disappear upon discontinuation of the IMP, yet an IMP relationship clearly exists.<br>• It follows a known pattern of response to the IMP.

5.2.5. Serious Adverse Events

5.2.5.1. Definition of a Serious Adverse Event

A serious adverse event is an adverse event that results in any of the following outcomes or actions:

- death
- a life threatening adverse event (ie, the subject was at immediate risk of death from the event as it occurred); does not include an event that, had it occurred in a more severe form, might have caused death
- inpatient hospitalization or prolongation of existing hospitalization, which means that hospital inpatient admission and/or prolongation of hospital stay were required for treatment of an adverse event, or that they occurred as a consequence of the event. Hospitalizations scheduled prior to study entry will not be considered serious adverse events, unless there was worsening of the preexisting condition during the subject’s participation in this study
- persistent or significant disability or incapacity (refers to a substantial disruption of one’s ability to conduct normal life functions)
- a congenital anomaly/birth defect
- an important medical event that may not result in death, be life-threatening, or require hospitalization, but may jeopardize the subject and may require medical intervention to prevent one of the outcomes listed in this definition. Examples of such events are intensive treatment in an emergency room or at home for allergic bronchospasm; blood dyscrasias or convulsions that do not result in hospitalization; or the development of drug dependency or drug abuse.

An adverse event that does not meet any of the criteria for seriousness listed above will be regarded as a nonserious adverse event.

5.2.5.2. Expectedness

A serious adverse event that is not included in the Adverse Reaction Section of the relevant reference safety information (RSI) by its specificity, severity, outcome, or frequency is considered an unexpected adverse event. The RSI for this study is the current IB for TV-46046, and US prescribing information for Depo- SubQ 104, Depo-Provera CI and Depo Provera 400 mg/mL (Depo-subQ 104 US prescribing information, Depo-Provera CI US prescribing information, and Depo-Provera Sterile Aqueous Suspension US prescribing information).

The sponsor's Pharmacovigilance Department will determine the expectedness for all serious adverse events.

For the purpose of SUSAR reporting, the version of the IB at the time of occurrence of the SUSAR applies.

5.2.5.3. Reporting a Serious Adverse Event

5.2.5.3.1. Investigator Responsibility

To satisfy regulatory requirements, all serious and protocol defined adverse events that occur during the study period (including the protocol defined follow-up period, regardless of judged relationship to treatment with the study drug), must be reported to the sponsor by the investigator. The event must be reported within 24 hours of when the investigator learns about it. Completing the serious and protocol defined adverse event form and reporting the event must not be delayed, even if not all the information is available.

The serious adverse event form will be sent to FHI 360 (contact information can be found in Appendix A). FHI 360 will forward the report to the sponsor's Global Patient Safety & Pharmacovigilance Department.

The following information should be provided to record the event accurately and completely:

- study number
- investigator and investigational center identification
- participant number
- onset date and detailed description of adverse event
- investigator's assessment of the relationship of the adverse event to the study drug (no reasonable possibility, reasonable possibility)

Additional information may include the following:

- age and sex of subject
- date of study drug injection
- action taken
- outcome, if known
- severity
- explanation of assessment of relatedness

- concomitant therapy (including doses, routes and regimens) and treatment of the event
  - pertinent laboratory or other diagnostic test data
  - medical history
  - for an adverse event resulting in death:
    - cause of death (whether or not the death was related to study drug)
    - autopsy findings (if available)

The investigator does not need to actively monitor subjects for adverse events once this study has ended. Serious adverse events occurring to a subject after the treatment of that subject has ended will be reported to the sponsor if the investigator becomes aware of them.

Each report of a serious adverse event will be reviewed and evaluated by the investigator and the sponsor to assess the nature of the event and the relationship of the event to the study drug, study procedures, and to underlying disease.

If additional (follow-up) information about a serious adverse event becomes available, the investigator will forward it to the sponsor within 24 hours.

Blinding will be maintained for the people who are directly involved in the study except for authorized unblinded study staff and the unblinded study monitor. In the case of a SUSAR, only the sponsor and a designated unblinded FHI 360 staff will receive the unblinded report for regulatory submission; the others will receive a blinded report.

5.2.5.3.2. Sponsor Responsibility

If a serious unexpected adverse event is believed to be related to the study drug or study procedures, the sponsor will take appropriate steps to notify all investigators participating in sponsored clinical studies of TV-46046 and the appropriate health authorities and IRB/IEC, if appropriate.

In addition to notifying the investigators and health authorities (and IRB/IEC, if appropriate), other measures may be required, including the following:

- amending the protocol
- discontinuing or suspending the study
- informing current study subjects of new findings by amending the existing ICF and re-consenting all subjects
- modifying listings of expected toxicities to include adverse events newly identified as related to TV-46046

5.2.6. Protocol Defined Adverse Events for Expedited Reporting

No protocol defined adverse events for expedited reporting to the sponsor were identified for this study.

5.2.7. Weight and Vital Signs

Weight and vital signs (BP [systolic/diastolic], temperature, pulse and respiration rate) will be measured at enrollment, Month 6, and study exit as detailed in Table 1.

Before pulse and BP are measured, the subject must be in a supine or semi-erect/seated position and resting for at least 5 minutes. (The same position and arm should be used each time vital signs are measured for a given subject.) For any abnormal vital sign finding, the measurement will be repeated. Any vital sign value that is judged by the investigator as a potentially clinically significant change (worsening) from a baseline value will be considered an adverse event. Weight gain or loss will be documented as an adverse event only if reported by the subject.

5.2.8. Medication Error and Special Situations Related to the Investigational Medicinal Products

Any administration of IMP that is not in accordance with the study protocol will be recorded as a deviation. If it meets the important protocol deviation criteria, the incorrect IMP administration will be categorized as "Non-Compliance to Investigational Medicinal Product (IMP)."

The following are types of medication errors and special situations:

- Medication error: Any unintentional error in the prescribing, dispensing, or administration of a medicinal product while in the control of the healthcare professional, patient, or consumer.
- Overdose: Administration of a quantity of a medicinal product given per administration or cumulatively which is above the maximum recommended dose according to the authorized product information. Clinical judgment should always be applied. Any dose of IMP (whether the test IMP, reference IMP, or placebo IMP), whether taken intentionally or unintentionally, in excess of that prescribed must be immediately reported to the sponsor.
- Misuse: Any intentional therapeutic use of a drug product in an inappropriate way or opioid use contrary to the directed or prescribed pattern of use, regardless of the presence or absence of harm or adverse effects. Examples: under usage, erratic or disorganized use, inappropriate use (for anxiety), in conjunction with alcohol or illegal substances, overuse.
- Abuse: Any intentional, nontherapeutic use of a drug product or substance, even once, for the purpose of achieving a desirable psychological or physiological effect, or intentional use of the opioid for a nonmedical purpose, such as euphoria or altering one's state of consciousness.
- Off-label use: Situations where an IMP is intentionally used for a medical purpose not in accordance with the authorized product information.
- Occupational exposure: Exposure to an IMP, as a result of one's professional or non-professional occupation.

5.2.9. Protocol Deviations Because of an Adverse Event

If a subject experiences an adverse event or medical emergency, departures from the protocol may be allowed on a case-by-case basis. After stabilization and/or treatment has been administered to ensure subject safety, the investigator or other physician in attendance must contact the physician identified in Appendix A of this protocol as soon as possible to discuss the situation. The investigator, in consultation with the sponsor and FHI 360, will decide whether the subject should continue to participate in the study.

5.2.10. Pregnancy

The risk of pregnancy is minimal since only women who are at low risk of pregnancy will be enrolled in the study (see Section 3.2.1 for Inclusion Criteria). Only women who are not pregnant on the day of the study injection and do not want to become pregnant in the next 18 months will be enrolled in the study. During the study, a urine pregnancy test will be performed if the woman is experiencing any symptoms or signs of pregnancy, or thinks she may be pregnant. In the unlikely event of pregnancy during the study, the subject will be withdrawn and pregnancy will be recorded as the reason for discontinuation on the appropriate study CRFs, and the investigator must provide FHI360 with the pregnancy form. The process for reporting a pregnancy is the same as that for reporting a serious adverse event (see Section 5.2.5.3 for details). All subjects who become pregnant will be monitored to the completion or termination of the pregnancy. If the pregnancy continues to term, the outcome, including spontaneous or voluntary termination, details of birth and presence or absence of any birth defect, congenital abnormalities, or maternal and newborn complications, will be reported to the study sponsor.

The investigator is not required to report subjects who are found to be pregnant between screening and baseline.

If the pregnancy does not continue to term, one of the following actions will be taken:

- For an elective abortion due to developmental anomalies, report as a serious adverse event and also fill in the pregnancy form.
- For an elective abortion **not** due to developmental anomalies, report on the pregnancy form; do not report as an adverse event.
- For a spontaneous abortion, report as a serious adverse event and also fill in the pregnancy form.

6. PHARMACOKINETIC MEASUREMENTS AND ASSESSMENTS

Pharmacokinetics will not be evaluated in this study.

7. PHARMACODYNAMIC, PHARMACOGENOMIC, IMMUNOGENICITY, AND BIOMARKER MEASUREMENTS AND ASSESSMENTS

7.1. Pharmacodynamics

Pharmacodynamics will not be evaluated in this study.

7.2. Pharmacogenomic Assessments

Pharmacogenomic sampling is not applicable in this study.

7.3. Immunogenicity

Immunogenicity will not be assessed in this study.

7.4. Assessment of Exploratory Biomarkers

Biomarkers will not be assessed in this study.

8. STATISTICS

This section describes statistical analyses of primary and secondary objectives as foreseen at the time the protocol was written. Further details will be described in a separate statistical analysis plan which will be approved prior to unblinding of study statisticians. Any additional analyses or changes to planned analyses will be fully disclosed in the clinical study report. All statistical analyses will be performed using SAS® software Version 9.4 or later. All tests and comparisons between treatment groups will be conducted at the two-sided 0.05 significance level, with no adjustment for multiple comparisons.

8.1. Sample Size and Power Considerations

The study size of 24 subjects, each of whom will receive all four treatments (one per abdominal quadrant), will provide at least 80% power to detect differences in the rates of ISRs among groups of the magnitude (>40%) observed in recent non-comparative studies of TV-46046 and Depo-subQ 104 (see Table 3). Although not designed to obtain precise estimates of less frequent ISR rates (eg, injection site discoloration [hypopigmentation]), there is a high (>90%) probability of detecting one or more specific ISR types per treatment if the true rate of such events is at least 10%. No randomized subjects will be replaced. However, if more than two subjects fail to receive all four injections or discontinue before Month 6, then additional subjects may be enrolled to help ensure that there are at least 22 subjects who complete the treatment sequence and are evaluable for at least 6 months.

Table 3: ISR Rates (per injection)

	Injection Pain	Redness	Injection Site Discoloration (Hypopigmentation)	Any Adverse Event	Reference
TV-46046 (Study #TV-46046-WH-10075; IND 126249)	8/12 (66.7%)	9/12 (75.0%)	2/12 (16.7%)	9/12 (75.0%)	Ongoing PK/PD
Depo-subQ 104 (Study #702179; IND 127097)	3 /18 (16.7%)	1/18 (5.5%)	0/18 (0%)	2/18 (11.1%)	Ongoing PK/PD

8.2. Method of Randomization

Each eligible subject will be randomized to one of the 24 possible treatment sequences (one subject per sequence). The randomization scheme will result in each drug being injected first, second, third, fourth on 6 occasions; and each drug being injected in each quadrant of the abdomen on 6 occasions.

Allocation assignments will be concealed using a centralized randomization application implemented within the OpenClinica data management system, with sequentially-numbered concealed envelopes available at the investigational center as a back-up method of assignment. The next available randomization sequence will be assigned to each subject only after she has been enrolled into the study. See Section 3.4.1 for how randomization will be performed and blinding maintained by the study staff.

8.3. Analysis Sets

There will be a single analysis set defined for this study: the Treated Analysis Set, which will include all subjects who receive at least 1 of the 4 study injections. All analyses will be performed according to treatment group, even if the result of a randomization or allocation error.

8.4. Data Handling Conventions

Any decision to exclude potential outliers will be made in blinded review. No missing data will be imputed.

8.5. Study Population

The set of randomized subjects who receive at least one injection will be used for all study population summaries, unless otherwise noted.

8.5.1. Subject Disposition

The number of screened and randomized subjects, number of subjects who fail to receive all four injections, total study visits completed and subject end of study status (eg, completed study, lost to follow-up, or discontinued before study completion) will be summarized using frequencies and percentages. Only women who are not enrolled will be considered screening failures. Reasons for early withdrawal will be listed.

8.5.2. Demographic and Baseline Characteristics

Subject demographic and baseline characteristics will be summarized for all subjects in the Treated Analysis Set. For continuous variables, descriptive statistics (number [n], mean, standard deviation, median, minimum, and maximum) will be provided. For categorical variables, subject counts and percentages will be provided. Categories of missing data will be presented if necessary.

8.6. Analysis of Primary Endpoints

8.6.1. Local Tolerability

The primary ISR endpoint will be assessed at least twice on Day 0: immediately after [ie, as soon as possible but no later than 10 minutes upon removing the needle] and again 1 hour after each of the four injections), at Days 1, 3, 7 and 14, at Months 3 and 6, and at additional visits, if indicated. Prior to the interim and/or final analysis, FHI 360's clinician will conduct a blinded review of ISR outcome information (recorded on study CRFs and clinical notes, if necessary) to assess adherence to protocol definitions; proper ISR and/or adverse event documentation and reporting; and potential redundant endpoints. All documented ISRs will be summarized for each treatment group using frequencies and percentages. The frequency of ISRs as well as ISRs that meet the per-protocol definition of an adverse event, will be compared between the treatment groups.

If there are sufficient numbers of ISRs to warrant the use of asymptotic methods, pair-wise treatment odds ratios for ISR (there are 6 possible comparisons among 4 treatment groups) will be assessed using logistic regression models with generalized estimating equations and robust

variance estimation to account for the correlated nature of the data. The primary model for such ISRs will only include effects of treatment group. An exchangeable working correlation structure will be used to estimate covariance parameters. Parameter estimates, standard errors, 95% confidence intervals, Z scores, and p-values for parameter estimates will also be produced. In addition, if the population is sufficiently heterogeneous with respect to these factors, exploratory analyses may be performed to assess effect of injection order, age and race. For less frequent ISR types, differences between groups will be assessed using exact McNemar's tests (separately for each of the 6 possible treatment comparisons). A final determination of whether the asymptotic method is appropriate will be made by the lead statistician based on observed correlation structures and event rates, blinded to treatment group prior to database lock and will be documented in the final statistical analysis plan. Regardless of analysis method, all tests will be conducted at the nominal two-sided 0.05 significance level with no adjustment for multiple comparisons.

8.7. Analysis of Secondary Endpoints

8.7.1. Injection Site Pain

NRS pain scores for assessments on the day of injection (Day 0) will be summarized by treatment group in frequency tables, separately for pain measured immediately after (ie, as soon as possible but no later than 10 minutes upon removing the needle) injection and at 1 hour after injection. If an appropriate distribution of pain scores can be identified in the blinded review (e.g., continuous, negative binomial or Poisson), then generalized linear models with exchangeable working correlation structure to account for correlated data will be used to compare treatment groups. The primary analysis model will only include effects of treatment, but exploratory analyses will be performed to assess effects of injection order, age, previous experience with injectable contraceptives, and race. If the dispersion of NRS scores is not amenable to parametric regression methods, then rank-order, permutation tests will be used to compare treatment groups (excluding any subjects who did not receive both injections being compared).

Approximately 1 hour after completing their sequence of the four treatments, each subject will also be asked to rank the injections according to overall pain from least [1] to most [4] painful. The ranking of treatments will be summarized in a frequency table, restricted to subjects who received all four injections. Differences in ranking of most painful injection will be assessed using an exact multinomial test for homogeneity across groups.

The proportion of subjects who self-report injection site pain during the follow-up will be compared between the treatment groups using exact, pair-wise McNemar tests

8.7.2. Overall Safety

Adverse events will be summarized in listings and frequency tables by treatment group according to system organ class, preferred term, and relatedness (noting it may not be possible to assess relatedness of systemic adverse events when all subjects receive all treatments). Medical coding will be done using WHO Drug. Concomitant medication use will also be summarized in frequency tables and subject listings. Change in weight and vital signs compared to baseline will

be summarized descriptively (no comparisons between treatment groups will be possible given that all subjects receive all four treatments at enrollment).

Additional details of all primary and secondary endpoint analyses (including table, figure, and listing shells), will be provided in the expanded statistical analysis plan.

9. INVESTIGATIONAL MEDICINAL PRODUCT INFORMATION

9.1. Investigational Medicinal Product Storage and Security

All IMPs (TV-46046, diluted TV-46046, TV-46046 Placebo, and Depo-subQ 104) must be stored according to the manufacturer's drug product stipulation, in a dry place, and in a securely locked, substantially constructed cabinet or enclosure. TV-46046 should be stored at controlled room temperature 20 to 25°C (68 to 77°F).

The investigator must confirm appropriate temperature conditions have been maintained for all IMPs received and any discrepancies are reported and resolved before use of the IMPs.

9.2. Investigational Medicinal Product Accountability

Each IMP shipment will include a packing slip listing the contents of the shipment and any applicable forms. The investigator is responsible for ensuring that deliveries of IMP and other study materials from the sponsor are correctly received, recorded, handled and stored safely and properly in accordance with the CFR or local regulations, and used in accordance with this protocol.

A record of IMP accountability (ie, IMP and other materials received, used, retained, returned, or destroyed) must be prepared and signed by the investigator, with an account given for any discrepancies. Empty and partially used containers of IMP will be destroyed at the investigational center in accordance with investigational center SOPs, with sponsor approval. In the event the investigational center is unable to destroy the empty and/or unused units of IMP, the IMP will be disposed of, retained, or returned to the sponsor or designee per FHI 360 instructions.

IMP accountability includes maintaining accurate records of quantity of products received, date of receipt, condition at receipt, temperature noted during transit, lot number of clinical study products received and dispensed, description of damaged units if any, dispensation, and product disposition or destruction. Storage temperature continuity tracking will occur for clinical study products during storage and transit time. Study personnel are responsible for daily temperature monitoring and safe storage of the study products.

10. QUALITY CONTROL AND QUALITY ASSURANCE

10.1. Protocol Amendments, Protocol Deviations, and Important Protocol Deviations

10.1.1. Protocol Amendments

No changes from the final approved (signed) protocol will be initiated without the prior written approval or favorable opinion of a written amendment by the IRB/IEC and local health authorities, as applicable, except when necessary to address immediate safety concerns to the subjects or when the change involves only nonsubstantial logistics or administration. The investigator, coordinating investigator and the sponsor will sign the protocol amendment.

10.1.2. Important Protocol Deviations

Any deviation from the protocol that affects, to a significant degree, (a) the safety, physical, or mental integrity of the subjects in the study and/or (b) the scientific value of the study will be considered an important protocol deviation. Important protocol deviations may include non-adherence on the part of the subject, the investigator, or the sponsor to protocol-specific inclusion and exclusion criteria, primary objective variable criteria, or GCP guidelines; or noncompliance to IMP administration. Important protocol deviations will be identified and recorded by study staff on a CRF and will be reported to the responsible IRB/IEC, as required.

When an important protocol deviation is reported, FHI 360 in consultation with the sponsor will determine whether to discontinue the subject from the study or permit the subject to continue in the study. The decision will be based on ensuring the safety of the subject and preserving the integrity of the study.

Changes in the inclusion and exclusion criteria of the protocol are not prospectively granted by the sponsor. If study staff learn that a subject who did not meet protocol inclusion and exclusion criteria was entered in a study, they must immediately inform FHI 360 of the important protocol deviation. If such a subject has already completed the study or has withdrawn early, no action will be taken but the important protocol deviation will be recorded.

FHI 360 will record lesser protocol deviations, ie those that do not significantly affect subject safety or scientific value of the data, on a Protocol Deviation Log. The cumulative log will be submitted to the responsible IRB/IEC at annual reviews, if/as required.

10.2. Study Monitoring

To ensure compliance with GCP guidelines, the study monitor or representative is responsible for ensuring that subjects have signed the ICF and the study is conducted in accordance with applicable SOPs, the protocol, and other written instructions and regulatory guidelines. Details of the monitoring procedures are outlined in the study's clinical monitoring plan which is maintained by FHI 360.

The main responsibilities of the study monitor are to ensure adherence to the protocol, that all data are correctly and completely recorded and reported, and that informed consent is obtained and recorded for all subjects before they participate in the study and when changes to the consent

form are warranted, in accordance with IRB/IEC approvals. The study monitor will be permitted to check and verify the various records (CRFs and other pertinent source data records), including specific electronic source documentation (see Section 12.1) relating to the study to verify adherence to the protocol and to ensure the completeness, consistency, and accuracy of the data being recorded.

As part of the supervision of study progress, other sponsor or FHI 360 personnel may, on request, accompany the study monitor on visits to the investigational center. The investigator and assisting staff must agree to cooperate with the study monitor to resolve any problems, errors, or possible misunderstandings concerning the findings detected during these monitoring visits and/or provided in follow-up written communication.

10.3. Clinical Product Complaints

A clinical product complaint is defined as a problem or potential problem with the physical quality or characteristics of clinical IMP supplies and/or clinical device supplies used in a sponsor's clinical research study. Examples of a product complaint include but are not limited to the following:

- suspected contamination
- questionable stability (eg, color change, flaking, crumbling, etc.)
- defective components
- missing or extra units (eg, primary container is received at the investigational center with more or less than the designated number of units inside)
- incorrect packaging or incorrect or missing labeling/labels
- unexpected or unanticipated taste or odor, or both
- device not working correctly or appears defective in some manner

Each investigational center will be responsible for reporting a possible clinical product complaint by completing the Product Complaint Form provided by the sponsor and emailing it to clinical.productcomplaints@tevapharm.com within 48 hours of becoming aware of the issue.

For complaints involving a device or other retrievable item, it is required that the device (or item) be sent back to the sponsor for investigative testing whenever possible. For complaints involving an IMP, all relevant samples (eg, the remainder of the subject's IMP supply) should be sent back to the sponsor for investigative testing whenever possible.

10.3.1. Product Complaint Information Needed from the Investigational Center

In the event that the Product Complaint Form cannot be completed, the investigator will obtain the following information, as available:

- investigational center number and investigator name
- name, phone number, and address of the source of the complaint
- clinical protocol number

- subject identifier (subject study number) and corresponding visit numbers, if applicable
- product name and strength for open label studies
- subject number, bottle, and kit numbers (if applicable) for double-blind or open label studies
- product available for return Yes/No
- product was taken or used in accordance with the protocol Yes/No
- description or nature of complaint
- associated serious adverse event Yes/No
- clinical supplies unblinded (for blinded studies) Yes/No
- date and name of person receiving the complaint

Note: Reporting a complaint must not be delayed even if not all the required information can be immediately obtained. Known information must be immediately reported. The sponsor will collaborate with the investigator to obtain any outstanding information.

10.3.2. Handling the Product Complaint IMP at the Investigational Center

The investigator is responsible for retaining the IMP related to the product complaint in a location separate from the investigator's clinical study supplies. The sponsor may request that the investigator return the product for further evaluation and/or analysis. If this is necessary, the clinical study monitor or designee will provide the information needed for returning the IMP.

If it is determined that the investigational center must return all IMP, the sponsor will provide the information needed to handle the return.

The integrity of the randomization code and corresponding blinded clinical supplies will be maintained whenever possible.

10.3.3. Documenting a Product Complaint

The investigator will record a description of the product complaint in the source documentation, along with any actions taken to resolve the complaint and to preserve the safety of the subject. Once the complaint has been investigated by the sponsor and the investigator, if necessary, an event closure letter may be sent to the investigational center where the complaint originated or to all investigational centers using the product.

10.4. Data Quality Control

The investigator is responsible for the accuracy, quality, completeness, and internal consistency of the data from this study. Data handling, including data quality control, will comply with international regulatory guidelines, including ICH GCP guidelines. Data management and control processes specific to this study, along with all steps and actions taken regarding data management and data quality control, will be described in a data management plan.

Data will be verified by the study monitor using the data source, and reviewed by Data Management using both automated logical checks and manual review. Data identified as erroneous, or data that are missing, will be referred to the investigational center for resolution through data queries. Any necessary changes will be made in the clinical database, and data review and validation procedures will be repeated as needed.

Case report forms will be processed and reviewed for completeness, consistency, and the presence of mandatory values. Applicable terms will be coded according to the coding conventions for this study. Logical checks will be implemented to ensure data quality and accuracy. Any necessary changes will be made in the clinical database, and data review and validation procedures will be repeated as needed. Data from external sources will be compared with the information available in the clinical data management system (CDMS). Discrepancies found will be queried.

Data corrections in the CDMS will be made using the CDMS update function. The system requires a reason for each change and keeps a complete audit trail of the data values, dates and times of modifications, and authorized electronic approvals of the changes.

At the conclusion of the study, the CDMS and all other study data will be locked to further additions or corrections. Locking the study data represents the acknowledgement that all data have been captured and confirmed as accurate. All data collected will be approved by the investigator at the investigational center.

10.5. Audit and Inspection

The sponsor or FHI 360 may audit the investigational center to evaluate study conduct and compliance with protocols, SOPs, GCPs, and applicable regulatory requirements. The sponsor's Global Clinical Quality Assurance department, independent of the Global Clinical Development department, is responsible for determining the need for (and timing of) an investigational center audit.

The investigator must accept that health authorities and sponsor representatives may conduct inspections to verify compliance with GCP guidelines.

11. ETHICAL AND REGULATORY CONSIDERATIONS

11.1. Investigator Responsibilities

This study will be conducted in full accordance with the International Council for Harmonisation (ICH) Harmonised Tripartite Guideline for Good Clinical Practice (GCP) E6 and any applicable national and local laws and regulations (eg, Title 21 Code of Federal Regulations [21CFR] Parts 11, 50, 54, 56, 312, and 314, Directive 2001/20/EC of the European Parliament and of the Council on the approximation of the laws, regulations and administrative provisions of the Member States relating to the implementation of GCP in the conduct of clinical studies on medicinal products for human use). Any episode of noncompliance will be documented.

The investigator has the overall responsibility for the conduct and administration of the clinical study and for contacts with study management, with the IRB/IEC, and with health authorities.

The investigator is responsible for performing the clinical study in accordance with this protocol and the applicable GCP guidelines referenced above for collecting, recording, and reporting the data accurately and properly. Agreement of the investigator to conduct and administer this clinical study in accordance with the protocol will be documented in separate clinical study agreements with FHI 360 and other forms as required by national health authorities in the country where each investigational center is located.

The investigator is responsible for ensuring the privacy, health, and welfare of the subjects during and after the clinical study; and must ensure that trained personnel are immediately available in the event of a medical emergency. The investigator and the involved clinical study personnel must be familiar with the background and requirements of the study; and with the properties of the IMPs as described in the IB or prescribing information.

The investigator is responsible for giving information about the study to all staff members involved in the study or in any element of subject management, both before starting the study and during the study (eg, when new staff become involved). The investigator must ensure that all study personnel are qualified by education, experience, and training to perform their specific responsibilities. These study personnel must be listed on the center's staff delegation log, which includes a clear description of each staff member's responsibilities and appropriately delegated significant study-related responsibilities. This list must be updated throughout the study, as necessary.

11.2. Health Authorities and Independent Ethics Committees/Institutional Review Boards

Before this study starts, the protocol will be submitted to health authorities, if required, and to each IRB/IEC for review. As required, the study will not start at Profamilia before the IEIRB/IEC and health authority (as applicable) for the center give written approval or a favorable opinion.

11.3. Informed Consent

The investigator, or a qualified person designated by the investigator, will fully inform the subject of all pertinent aspects of the study, including the written information approved by the IRB/IEC. All written and oral information about the study will be provided in a language as nontechnical as practical and understood by the subject. The subject will be given ample time and opportunity to inquire about details of the study and to decide whether to participate in the study. The above will be detailed in the source documentation.

Written informed consent will be obtained from each subject before any study specific procedures or assessments are done and after the aims, methods, anticipated benefits, and potential hazards are explained, in accordance with applicable regulatory requirements. The subject's willingness to participate in the study will be documented in an ICF, which will be signed and personally dated by the subject and by the person who conducted the informed consent discussion. The investigator will keep the original consent forms, and copies will be given to the subject. It will also be explained to the subject that the subject is free to refuse entry into the study and free to withdraw from the study at any time without prejudice to future treatment.

11.4. Subject Confidentiality

The investigator must ensure that the privacy of the subjects, including their identity and all personal medical information, will be maintained at all times. In CRFs and other documents or image material submitted to the sponsor, subjects will be identified not by their names, but by an identification code (ie, identification number).

Personal medical information may be reviewed for subject safety and for verifying data in the source and transcribed onto the CRF. This review may be conducted by the study monitor, properly authorized persons on behalf of the sponsor or FHI 360, Global Quality Assurance (GQA), or health authorities. Personal medical information will always be treated as confidential.

11.5. Declaration of the End of the Clinical Study

Local regulations will be followed to determine the end of the study.

11.6. Registration of the Clinical Study

In compliance with local regulations and in accordance with the sponsor's standard procedures, this clinical study will be registered on clinicaltrials.gov.

12. STUDY DOCUMENTATION

12.1. Source Data and Case Report Forms

Data will be collected at the investigational center by appropriately designated and trained personnel. The investigator must maintain the original records (ie, source documents) of each subject's data at all times. The investigator will maintain a confidential subject identification list that allows the unambiguous identification of each subject. Subject identity will not be discernible from the data provided on CRFs.

Examples of source documents are hospital records, office visit records, examining physician's finding or notes, laboratory reports, drug inventory, IMP label records, and worksheets that are used as the source.

Some data may be recorded directly onto the CRF, if instructed by FHI 360; the investigational center will complete a Source Data Guide document specifying which data are recorded directly onto CRFs. "Case report form" means any CRF, whether paper or electronic.

The medical experts, study monitors, auditors, IRB/IEC, and inspectors from health authorities (or their agents) will be given direct access to source data and documents (eg, medical charts/records, laboratory test results, printouts) for source data verification, provided that subject confidentiality is maintained in accordance with national and local requirements.

All data collected will be approved by the investigator at the investigational center. This approval acknowledges the investigator's review and acceptance of the data as being complete and accurate.

The data collected on CRFs will be entered in a clinical data management system (CDMS) that meets the technical requirements described in 21CFR Part 11 (USA) and documents of other concerned health authorities. The CDMS will be fully validated to ensure that it meets the scientific, regulatory, and logistical requirements of the study before it is used to capture data from this study. Before using the CDMS, all users will receive training on the system and study specific training. After they are trained, users will be provided with individual system access rights.

For subjects who sign an ICF but do not meet eligibility criteria, at a minimum, data for screen failure reason, demography, and adverse events from the time of informed consent will be entered onto a CRF.

12.2. Archiving of Study Documentation

12.2.1. FHI 360 Responsibilities

All data management tasks for this study are delegated to FHI 360; these functions will be carried out as described in FHI 360 SOPs. The original CRFs will be stored at the respective investigational centers until the end of the study.

12.2.2. Investigator Responsibilities

The investigator must maintain all written and electronic records, accounts, notes, reports, and data related to the study and any additional records required to be maintained under country, state/province, or national and local laws, including, but not limited to:

- full case histories
- signed ICFs
- subject identification lists
- case report forms for each subject on a per-visit basis
- data from other sources (eg, external laboratory)
- safety reports
- reports of receipt, use, and disposition of the IMPs
- copies of all correspondence with FHI 360, the IRB/IEC, and any health authority

The investigator will retain all records related to the study and any additional records required, as indicated by the protocol and according to applicable laws and regulations, until FHI 360 or sponsor notifies the institution in writing that records may be destroyed. If, after 25 years from study completion, or earlier in the case of the investigational center closing or going out of business, the investigator reasonably determines that study record retention has become unduly burdensome, and FHI 360 has not provided written notification of destruction, then the investigator may submit a written request to FHI 360 at least 60 days before any planned disposition of study records. After receipt of such request, FHI 360 may arrange for appropriate archival or disposition, including requiring that the investigator deliver such records to the sponsor or FHI 360. The investigator shall notify FHI 360 of any accidental loss or destruction of study records.

13. FINANCING AND INSURANCE

A separate clinical study agreement, including a study budget, will be entered into between each investigator and FHI 360 before the study drug is delivered.

This clinical study is insured in accordance with the corresponding local legal provisions. The policy coverage is subject to the full policy terms, conditions, extensions, and exclusions. Excluded from the insurance coverage are inter alia, damages to health, and worsening of previous existing disease that would have occurred or continued if the subject had not taken part in the clinical study.

14. REPORTING AND PUBLICATION OF RESULTS

The sponsor is responsible for ensuring that the public has access to the appropriate information about the study by conforming to local and regional requirements and regulations for registration and posting of results, and to requirements outlined in any current or future agreements between the sponsor and FHI 360.

FHI 360 will prepare the clinical study report, in cooperation with the sponsor. The final report is signed by the sponsor and FHI 360.

When the sponsor generates reports from the data collected in this study for presentation to health authorities, drafts will be circulated to FHI 360 for comments and suggestions.

No unpublished information shall be published or disclosed to a third party without the prior written consent of the sponsor and FHI 360. The primary publication from this study will report the results of the study in accordance with the current "Recommendations for the Conduct, Reporting, Editing, and Publication of Scholarly Work in Medical Journals" (www.ICMJE.org). Publication of the results will occur in a timely manner according to applicable regulations. Authorship will be based on meeting all the following 4 criteria:

- substantial contributions to the conception or design of the work; or the acquisition, analysis, or interpretation of data for the work
- drafting the work or revising it critically for important intellectual content
- final approval of the version to be published
- agreement to be accountable for all aspects of the work in ensuring that questions related to the accuracy or integrity of any part of the work are appropriately investigated and resolved.

A joint publications committee will be established by the sponsor and FHI 360 to oversee this process. Additional publications may follow upon the agreement of the committee. Policies regarding the publication of the study results are further defined in a collaboration agreement between the sponsor and FHI 360. Any disputes or issues about publication will be referred to the Executive Committee, as described in that agreement.

No patent applications based on the results of the study may be made by the investigator nor may assistance be given to any third party to make such an application without the written authorization of the sponsor.

15. REFERENCES

Castle WM, Sapire KE, Howard KA. Efficacy and acceptability of injectable medroxyprogesterone. A comparison of 3-monthly and 6-monthly regimens. S Afr Med J. 1978;53(21):842-5.

Curtis KM, Tepper NK, Jatlaoui TC. U.S. Medical Eligibility Criteria for Contraceptive Use, 2016. MMWR Recomm Rep 2016; 65(No. RR-3):35-54. Appendices available at: https://www.cdc.gov/mmwr/volumes/65/rr/pdfs/rr6503.pdf. Accessed on 04 May 2018

Depo subQ Provera 104 [package insert]. New York, NY: Pfizer Inc; 2016.

Depo-Provera Sterile Aqueous Suspension US prescribing information [package insert]. New York, NY: Pfizer Inc; 2017.

Depo-Provera Contraceptive Injection [package insert]. New York, NY: Pfizer Inc; 2016.

Dias C, Abosaleem B, Crispino C, Gao B, Shaywitz A. Tolerability of High-Volume Subcutaneous Injections of a Viscous Placebo Buffer: A Randomized, Crossover Study in Healthy Subjects. AAPS PharmSciTech. 2015 Oct;16(5):1101-7. Epub 2015 Feb 19. Erratum in: AAPS PharmSciTech. 2015 Dec;16(6):1500

Jain J, Dutton C, Nicosia A, Wajszczuk C, Bode FR, Mishell DR Jr. Pharmacokinetics, ovulation suppression and return to ovulation following a lower dose subcutaneous formulation of Depo-Provera. Contraception. 2004;70(1):11-8.

Kaunitz AM, Darney PD, Ross D, Wolter KD, Speroff L. Subcutaneous DMPA vs. intramuscular DMPA: a 2-year randomized study of contraceptive efficacy and bone mineral density. Contraception. 2009;80:7-17.

Kaunitz AM, Grimes DA. Removing the black box warning for depot medroxyprogesterone acetate. Contraception. 2011;84(3):212-3.

Lee NC, Rosero-Bixby L, Oberle MW, Grimaldo C, Whatley AS, Rovira EZ. A case-control study of breast cancer and hormonal contraception in Costa Rica. J Natl Cancer Inst. 1987;79(6):1247-54.

Li CI, Beaber EF, Tang MTC, Porter PL, Daling JR, Malone KE. Effect of depo-medroxyprogesterone acetate on breast cancer risk among women 20 to 44 years of age. Cancer Res. 2012;72(8):2028-35.

Mackay EV, Khoo SK, Adam RR. Contraception with a six-monthly injection of progestogen. Part 1. Effects on blood pressure, body weight and uterine bleeding pattern, side-effects, efficacy, and acceptability. Aust and N Z J Obstet Gynaecol. 1971;11(3):148-55.

McDaniel EB, Pardthaisong T. Use-effectiveness of six-month injections of DMPA as a contraceptive. Am J of Obstet and Gynecol. 1974; 119(2):175-80.

Morrison CS, Chen P-L, Kwok C, Baeten JM, Brown J, Crook AM, et al. Hormonal contraception and the risk of HIV acquisition: An individual participant data meta-analysis. PLoS medicine. 2015;12(1):e1001778.

Paul C, Skegg DCG, Spears GFS. Depot medroxyprogesterone (Depoi-Provera) and risk of breast cancer. Br Med J 1989:299(6702);759-62.

Polis CB, Curtis KM, Hannaford PC, Phillips SJ, Chipato T, Kiarie JN, et al. An updated systematic review of epidemiological evidence on hormonal contraceptive methods and HIV acquisition in women. AIDS. 2016;30(17):2665-83.

PRDEPO-PROVERA and PRDEPO-PROVERA-SC [product monograph]. Kirkland, Quebec: Pfizer Canada, Inc; 2013.

Ralph LJ, McCoy SI, Shiu K, Padian NS. Hormonal contraceptive use and women's risk of HIV acquisition: a meta-analysis of observational studies. Lancet. 2015;15(2):181-9.

Schwallie PC, Assenzo JR. Contraceptive use--efficacy study utilizing Depo-Provera administered as an injection once every six months. Contraception. 1972;6(4):315-27.

Shapiro S, Rosenberg L, Hoffman M, Truter H, Cooper D, Rao S, et al. Risk of breast cancer in relation to the use of injectable progestogen contraceptives and combined estrogen/progestogen contraceptives. Am J Epidemiol 2000;151(4);396-403.

World Health Organization Department of Reproductive Health and Research. Hormonal contraceptive eligibility for women at high risk of HIV: 2017 guidance statement (WHO/RHR/17.04). Geneva: World Health Organization; 2017. Available at: http://www.who.int/reproductivehealth/publications/family_planning/HC-and-HIV-2017/en/. Accessed on 26 March 2018.

World Health Organization. Breast cancer and depot-medroxyprogesterone acetate: a multinational study. Lancet. 1991;338(8771):833-8.

16. SUMMARY OF CHANGES TO PROTOCOL

16.1. Protocol Amendment 04 Dated 18 May 2020

The primary reason this protocol amendment are administrative updates and to update the procedures after the follow-up period and before the close out monitoring visit.

Original text with changes shown	New wording	Reason/Justification for change
**COVER PAGE**		
**Sponsor**<br>Teva Branded Pharmaceutical Products R&D, Inc.<br>~~41 Moores Road~~<br>~~Frazer, Pennsylvania 19355~~<br>~~United States~~<br>145 Brandywine Pkwy,<br>West Chester, PA 19380<br>United States	**Sponsor**<br>Teva Branded Pharmaceutical Products R&D, Inc.<br>145 Brandywine Pkwy,<br>West Chester, PA 19380<br>United States	Address updated.
**Authorized Representative**<br>Alexandra Kropotova, MD, MBA<br>Vice President, Global Specialty R&D, Respiratory TA Head<br>Teva Branded Pharmaceutical Products<br>R&D, Inc.<br>~~41 Moores Road~~<br>~~Frazer, Pennsylvania 19355~~<br>~~United States of America~~<br>~~+1-610-893-1145~~<br>145 Brandywine Pkwy,<br>West Chester, PA 19380<br>United States<br>+1-610-738-6557	**Authorized Representative**<br>Alexandra Kropotova, MD, MBA<br>Vice President, Global Specialty R&D, Respiratory TA Head<br>Teva Branded Pharmaceutical Products<br>R&D, Inc.<br>145 Brandywine Pkwy,<br>West Chester, PA 19380<br>United States<br>+1-610-738-6557	Address updated.
**Section 3.4.5. Post-6 Month Follow-up**		
~~If the subject does not have any ISRs at this visit, it will be her final visit and the subject will be instructed to contact the clinic if any ISRs develop after her final visit.~~ If any ISRs are identified or still ongoing at this additional visit between Months 9 and 12, monthly follow-up visits for ISR evaluation will be scheduled until the ISR is resolved or Month 18, whichever is earlier. In this case, ISR, adverse event, and concomitant medication evaluation, and vital sign and weight measurements will be repeated at her final visit.	If any ISRs are identified or still ongoing at this additional visit between Months 9 and 12, monthly follow-up visits for ISR evaluation will be scheduled until the ISR is resolved or Month 18, whichever is earlier. In this case, ISR, adverse event, and concomitant medication evaluation, and vital sign and weight measurements will be repeated at her final visit.	Update.

Original text with changes shown	New wording	Reason/Justification for change
If the subject reports injection site pain at any of these visits, she will be asked to use the NRS to evaluate the intensity of pain in order to determine if it meets the definition of an adverse event. If the subject does not have any ISRs at this visit, it will be her final visit. However, the subject will be instructed to return to the clinic if any ISRs develop after her final visit. In that case, she will be consented, her status will change from completed the study to active and she will be followed until ISR resolution or Month 18, whichever is earlier, as described above.	If the subject reports injection site pain at any of these visits, she will be asked to use the NRS to evaluate the intensity of pain in order to determine if it meets the definition of an adverse event. If the subject does not have any ISRs at this visit, it will be her final visit. However, the subject will be instructed to return to the clinic if any ISRs develop after her final visit. In that case, she will be consented, her status will change from completed the study to active and she will be followed until ISR resolution or Month 18, whichever is earlier, as described above.	Update.

16.2. Protocol Amendment 03 Dated 19 November 2019

The primary reasons for this amendment are updates, clarifications, and corrections. Changes made within the text of the protocol were also made within the synopsis.

Original text with changes shown	New wording	Reason/Justification for change
**TITLE PAGE**		
Authorized Representative<br>Alexandra Kropotova, MD, MBA<br>Vice President, Global Specialty R&D, Respiratory TA Head<br>Teva Branded Pharmaceutical Products R&D, Inc.<br>~~2 West Liberty Blvd, Suite 300~~<br>~~Malvern~~41 Moores Road<br>Frazer, Pennsylvania 19355<br>United States of America<br>+1-610-893-1145	Authorized Representative<br>Alexandra Kropotova, MD, MBA<br>Vice President, Global Specialty R&D, Respiratory TA Head<br>Teva Branded Pharmaceutical Products R&D, Inc.<br>41 Moores Road<br>Frazer, Pennsylvania 19355<br>United States of America<br>+1-610-893-1145	Correction.
Sponsor's Safety Representative<br>Dana Ilic Bogojevic, MD<br>Safety Physician, Medical Scientific Unit – US<br>Teva Branded Pharmaceutical Products R&D, Inc.<br>400 Interpace Pkwy, Bldg. A, Suite ~~221~~367<br>Parsippany, NJ 07054<br>+1-973-658-0378	Sponsor's Safety Representative<br>Dana Ilic Bogojevic, MD<br>Safety Physician, Medical Scientific Unit – US<br>Teva Branded Pharmaceutical Products R&D, Inc.<br>400 Interpace Pkwy, Bldg. A, Suite 367<br>Parsippany, NJ 07054<br>+1-973-658-0378	Correction.
**CLINICAL STUDY PROTOCOL SYNOPSIS**		
All subjects will be followed for at least 6 months after receiving their injections. ~~Subjects with injection site reactions (ISRs) will be followed through the resolution of ISR(s) or 18 months after injection, whichever comes first.~~	All subjects will be followed for at least 6 months after receiving their injections.	Correction. Duplicate sentence removed.
**Section 1.3 Known and Potential Benefits and Risks**		
~~Additional information regarding risks to subjects may be found in the current IB.~~ In addition to the benefit and risk information described in this section, please refer to the current IB (Section 3, Section 6.3, and Section 7).	In addition to the benefit and risk information described in this section, please refer to the current IB (Section 3, Section 6.3, and Section 7).	Clarification.
**Section 1.3.2.9 Other**		
Additional information regarding risks to subjects related to Depo-subQ 104 may be found in the ~~Summary of Product Characteristics (SPC)~~ Depo-subQ 104 Prescribing Information (Depo-subQ 104 US Prescribing Information 2016).	Additional information regarding risks to subjects related to Depo-subQ 104 may be found in the Depo-subQ 104 Prescribing Information (Depo-subQ 104 US Prescribing Information 2016).	Correction.

Original text with changes shown	New wording	Reason/Justification for change
**Section 1.4.1 General Study Design Rationale**		
In the ongoing TV46046-WH-10147 study, several cases of hypopigmentation had a later onset (ie, approximately 6 months post-injection) than observed in Study TV46046-WH-10075. In order to identify any late onset ISRs, subjects who have exited the study at Month 6 with no new or ongoing ISRs and who agree to return for a single follow-up visit between Months 9 and 12, will undergo ISR, adverse event, and concomitant medication evaluation, and vital sign and weight measurements at that time (see Section 3.4.5 for more detail).	In the ongoing TV46046-WH-10147 study, several cases of hypopigmentation had a later onset (ie, approximately 6 months post-injection) than observed in Study TV46046-WH-10075. In order to identify any late onset ISRs, subjects who have exited the study at Month 6 with no new or ongoing ISRs and who agree to return for a single follow-up visit between Months 9 and 12, will undergo ISR, adverse event, and concomitant medication evaluation, and vital sign and weight measurements at that time (see Section 3.4.5 for more detail).	Addition. Text added to explain why the duration of the follow-up period was extended.
**Section 3.1 General Study Design**		
Subjects with unresolved ISR(s) ~~at Month 6~~ will be followed monthly through the resolution of ISR(s) or Month 18, whichever comes first.	Subjects with unresolved ISR(s) will be followed monthly through the resolution of ISR(s) or Month 18, whichever comes first.	Clarification. Text edited to reflect extension of follow-up period.
Vital signs and weight will be measured at baseline, Month 6, and study exit.	Vital signs and weight will be measured at baseline, Month 6, and study exit.	Clarification. Text added to match Study Procedures and Assessments table.
**Table 1:Study Procedures and Assessments**		
Months 3 and 6 [a]	Months 3 and 6 [a]	Addition. Footnote added to describe subject's visits if ISRs are reported at 6 months.
Months 7, 8, 9, 10, 11, 12, 13, 14, 15, 16, 17, 18[b]	Months 7, 8, 9, 10, 11, 12, 13, 14, 15, 16, 17, 18[b]	Addition. Footnote added to describe study visits for those who have exited the study with no new or ongoing ISRs at Month 6.
[a] If the subject does not have any ISRs at Month 6 and does not return for an additional post-6 month follow up visit, Month 6 will be her final visit. In addition to ISR, adverse event, and concomitant medication evaluation, her weight and vital signs will be measured at that time. Subjects who complete the study at Month 6 will be instructed to contact the clinic if any ISRs develop after their final visit. If the subject is reported to have ISR(s) at Month 6, she will be instructed to return to the clinic monthly until ISR resolution or Month 18, whichever is earlier. In this case, the Month 6 procedures will be repeated at her final visit.	[a] If the subject does not have any ISRs at Month 6 and does not return for an additional post-6 month follow up visit, Month 6 will be her final visit. In addition to ISR, adverse event, and concomitant medication evaluation, her weight and vital signs will be measured at that time. Subjects who complete the study at Month 6 will be instructed to contact the clinic if any ISRs develop after their final visit. If the subject is reported to have ISR(s) at Month 6, she will be instructed to return to the clinic monthly until ISR resolution or Month 18, whichever is earlier. In this case, the Month 6 procedures will be repeated at her final visit.	Addition. Text edited to reflect extension of follow-up period.

Original text with changes shown	New wording	Reason/Justification for change
Subjects who have exited the study with no new or ongoing ISRs at Month 6 and who agree (providing written informed consent) to return for a single follow-up visit between Months 9 and 12, will undergo ISR, adverse event, and concomitant medication evaluation, and vital sign and weight measurements at that time. If the subject does not have any ISRs at this visit, it will be her final visit and the subject will be instructed to contact the clinic if any ISRs develop after their final visit. If the subject does have ISR(s) at this visit, she will be instructed to return to the clinic monthly until the ISR resolution or Month 18, whichever is earlier. In this case, ISR, adverse event, and concomitant medication evaluation, and vital sign and weight measurements will be repeated at her final visit.	Subjects who have exited the study with no new or ongoing ISRs at Month 6 and who agree (providing written informed consent) to return for a single follow-up visit between Months 9 and 12, will undergo ISR, adverse event, and concomitant medication evaluation, and vital sign and weight measurements at that time. If the subject does not have any ISRs at this visit, it will be her final visit and the subject will be instructed to contact the clinic if any ISRs develop after their final visit. If the subject does have ISR(s) at this visit, she will be instructed to return to the clinic monthly until the ISR resolution or Month 18, whichever is earlier. In this case, ISR, adverse event, and concomitant medication evaluation, and vital sign and weight measurements will be repeated at her final visit.	Addition. Text added to detail the follow-up visit between Months 9 and 12.
[c] Vital signs and weight will be measured on Enrollment/Injection Day (Day 0) and Month 6, and at study exit ~~(if different from Month 6)~~.	[c] Vital signs and weight will be measured on Enrollment/Injection Day (Day 0) and Month 6, and at study exit.	Addition. Weight assessment added to Day 0.
~~[e]If the subject does not have any ISRs at Month 6, Month 6 will be her final visit~~.	[Text removed]	Correction. Footnote removed.
[g] Study staff will inquire about adverse events and concomitant medications at Months 1, 2, 3, 4, 5 and 6 and at study exit, but will document adverse events and concomitant medications at any visit where the subject reports any new or changed adverse events or concomitant medications. For subjects who have previously exited the study at Month 6 and who agree to return for a single follow-up visit between Months 9 and 12, study staff will inquire about adverse events and concomitant medications during this visit and at study exit.	[g] Study staff will inquire about adverse events and concomitant medications at Months 1, 2, 3, 4, 5 and 6 and at study exit, but will document adverse events and concomitant medications at any visit where the subject reports any new or changed adverse events or concomitant medications. For subjects who have previously exited the study at Month 6 and who agree to return for a single follow-up visit between Months 9 and 12, study staff will inquire about adverse events and concomitant medications during this visit and at study exit.	Addition. Text added to describe adverse events inquires for subjects who have previously exited the study at Month 6 and who agree to return for a single follow-up visit between Months 9 and 12
**Section 3.4.3 Follow-up/Final Visits**		
If the subject does not have any ISRs at Month 6, Month 6 will be her final visit~~.~~, unless she agrees to return for additional follow up as detailed in Section 3.4.5.	If the subject does not have any ISRs at Month 6, Month 6 will be her final visit, unless she agrees to return for additional follow up as detailed in Section 3.4.5.	Addition. Text edited to reflect extension of follow-up period.
• injection sites evaluated for ongoing and/or new ISRs (Days 1, 3, 7, and 14 and Months 3 and 6 and at study exit)	• injection sites evaluated for ongoing and/or new ISRs (Days 1, 3, 7, and 14 and Months 3 and 6 and at study exit)	Clarification. Text added to describe when injection sites will be assessed.
• injection site pain assessed if self-reported (Days 1, 3, 7, and 14 and Months 3 and 6, and other visits if appropriate)	• injection site pain assessed if self-reported (Days 1, 3, 7, and 14 and Months 3 and 6, and other visits if appropriate)	Clarification. Text added to describe when injection site pain will be assessed.

**Original text with changes shown**	**New wording**	**Reason/Justification for change**
• adverse event and concomitant medication inquiry conducted (at Months 1, 2, 3, 4, 5 and 6 and at study exit)	• adverse event and concomitant medication inquiry conducted (at Months 1, 2, 3, 4, 5 and 6 and at study exit)	Clarification. Text added to describe when adverse event and concomitant medication inquiry will occur.
• vital signs and weight measured (Month 6 and at study exit)	• vital signs and weight measured (Month 6 and at study exit)	Clarification. Text added to describe when vital signs and weight will be measured.
**Section 3.4.5 Post-6 Month Follow-up**		
In order to identify any late onset ISRs, subjects who have exited the study with no new or ongoing ISRs at Month 6 and who agree (providing written informed consent) to return for a single follow-up visit between Months 9 and 12, will undergo ISR, adverse event and concomitant medication evaluation, and vital sign and weight measurements at that time. If the subject does not have any ISRs at this visit, it will be her final visit and the subject will be instructed to contact the clinic if any ISRs develop after her final visit. If any ISRs are identified or still ongoing at Month 9, monthly follow-up visits for ISR evaluation will be scheduled until the ISR is resolved or Month 18, whichever is earlier. In this case, ISR, adverse event, and concomitant medication evaluation, and vital sign and weight measurements will be repeated at her final visit. If the subject reports injection site pain at any of these visits, she will be asked to use the NRS to evaluate the intensity of pain in order to determine if it meets the definition of an adverse event.	In order to identify any late onset ISRs, subjects who have exited the study with no new or ongoing ISRs at Month 6 and who agree (providing written informed consent) to return for a single follow-up visit between Months 9 and 12, will undergo ISR, adverse event and concomitant medication evaluation, and vital sign and weight measurements at that time. If the subject does not have any ISRs at this visit, it will be her final visit and the subject will be instructed to contact the clinic if any ISRs develop after her final visit. If any ISRs are identified or still ongoing at Month 9, monthly follow-up visits for ISR evaluation will be scheduled until the ISR is resolved or Month 18, whichever is earlier. In this case, ISR, adverse event, and concomitant medication evaluation, and vital sign and weight measurements will be repeated at her final visit. If the subject reports injection site pain at any of these visits, she will be asked to use the NRS to evaluate the intensity of pain in order to determine if it meets the definition of an adverse event.	Addition. Section added to describe the post 6 month follow-up visits.
**Section 4.3 Blinding/Unblinding**		
~~All~~ FHI 360 ~~staff (aside from the unblinded clinical monitor)~~ and sponsor ~~staff~~ project team members involved in assessment of adverse events, data analysis, or results interpretation will be blinded to treatment sequence until at least the first interim analysis, at which time results may be reviewed by treatment to inform decisions whether to stop enrollment or modify the study.	FHI 360 and sponsor project team members involved in assessment of adverse events, data analysis, or results interpretation will be blinded to treatment sequence until at least the first interim analysis, at which time results may be reviewed by treatment to inform decisions whether to stop enrollment or modify the study.	Clarification. Details added to describe which study personnel will be blinded.
**Section 5.1.1 Local Tolerability**		

Original text with changes shown	New wording	Reason/Justification for change
~~All ongoing ISRs will be examined during the next clinic visit, regardless of visit type, or more frequently at the discretion of the investigator, until ISR resolution, or outcome. Subjects with no ISRs at the Month 6~~ Subjects with no new or ongoing ISRs at their final visit will complete the study but be instructed to contact the clinic if new ISRs develop after their final visit~~.~~ (see Section 3.4.3 and Section 3.4.5).	Subjects with no new or ongoing ISRs at their final visit will complete the study but be instructed to contact the clinic if new ISRs develop after their final visit (see Section 3.4.3 and Section 3.4.5).	Addition. Text added to describe procedures if new ISRs develop during the follow-up period.
Subjects with ISRs at ~~Month 6~~ their final visit will be followed monthly ~~(or more frequently if indicated)~~ until ISR resolution or Month 18, whichever comes first.	Subjects with ISRs at their final visit will be followed monthly until ISR resolution or Month 18, whichever comes first.	Addition. Text added to describe procedures if new ISRs develop during the follow-up period.
**Section 5.2 Safety Assessments**		
• vital signs and weight will be measured at Day 0 and Month 6 and study exit ~~(if not Month 6)~~	• vital signs and weight will be measured at Day 0 and Month 6 and study exit	Correction. Text removed.
• adverse events and concomitant medications will be evaluated at Day 0, ~~Month~~ at Months 1~~, and then monthly~~ through ~~Month~~ 6, and at study exit	• adverse events and concomitant medications will be evaluated at Day 0, at Months 1 through 6, and at study exit	Clarification. Text added to state that safety will be evaluated in the follow-up period.
**Section 5.2.2 Recording and Reporting Adverse Events**		
At the ~~Month 3~~ Day 0, Months 1 through 6 and ~~Month 6 follow up visits~~ study exit, the investigator must question the subject about adverse events by asking an open-ended question such as, "Have you had any unusual symptoms or medical problems since the last visit? If yes, please describe."	At the Day 0, Months 1 through 6 and study exit, the investigator must question the subject about adverse events by asking an open-ended question such as, "Have you had any unusual symptoms or medical problems since the last visit? If yes, please describe."	Addition. Details added to describe how adverse event data will be collected in the follow-up period.
**Section 5.2.7 Weight and Vital Signs**		
Weight and vital signs (BP [systolic/diastolic], temperature, pulse and respiration rate) will be measured at enrollment, ~~and~~ Month 6, and study exit as detailed in Table 1.	Weight and vital signs (BP [systolic/diastolic], temperature, pulse and respiration rate) will be measured at enrollment, Month 6, and study exit as detailed in Table 1.	Addition. Text added to describe weight and vital sign collection in follow-up period.
**Section 8.7 Analysis of Secondary Endpoints**		
**Section 8.7.2. Overall Safety**		
Change ~~from baseline to Month 6 visits~~ in weight and vital signs compared to baseline will be summarized descriptively (no comparisons between treatment groups will be possible given that all subjects receive all four treatments at enrollment).	Change in weight and vital signs compared to baseline will be summarized descriptively (no comparisons between treatment groups will be possible given that all subjects receive all four treatments at enrollment).	Clarification. Month 6 visits removed.
**Appendix A. STUDY RESPONSIBILITIES**		

Original text with changes shown	New wording	Reason/Justification for change
Dana Ilic Bogojevic, MD<br>Safety Physician, Medical Scientific Unit – US<br>Teva Branded Pharmaceutical Products R&D, Inc.<br>400 Interpace Pkwy, Bldg. A, Suite ~~221~~ 367<br>Parsippany, NJ 07054<br>+1-973-658-0378	Dana Ilic Bogojevic, MD<br>Safety Physician, Medical Scientific Unit – US<br>Teva Branded Pharmaceutical Products R&D, Inc.<br>400 Interpace Pkwy, Bldg. A, Suite 367<br>Parsippany, NJ 07054<br>+1-973-658-0378	Correction.

16.3. Protocol Amendment 02 Dated 11 April 2019

The primary reasons for this amendment are corrections.

Original text with changes shown	New wording	Reason/Justification for change
**Title Page**		
~~Claudio Pasquinelli MD, PhD~~<br>~~Sr Dir Clinical Development, Respiratory~~<br>~~Teva Branded Pharmaceutical Products R&D, Inc.~~<br>~~+1-610-893-1264~~<br>Michael J. Lamson, PhD<br>VP, PK and ClinPharm<br>Nuventra, Inc.™<br>2525 Meridian Parkway, Suite 280<br>Durham, North Carolina 27713<br>+1-919-667-7976	Michael J. Lamson, PhD<br>VP, PK and ClinPharm<br>Nuventra, Inc.™<br>2525 Meridian Parkway, Suite 280<br>Durham, North Carolina 27713<br>+1-919-667-7976	Correction. Updated staff information.
~~Haixiao Chen, MD~~<br>~~Global Patient Safety & Pharmacovigilance~~<br>~~Teva Branded Pharmaceutical Products R&D, Inc.~~<br>~~+1-610-893-1035~~<br>Dana Ilic Bogojevic, MD<br>Safety Physician, Medical Scientific Unit – US<br>Teva Branded Pharmaceutical Products R&D, Inc.<br>400 Interpace Pkwy, Bldg. A, Suite 221<br>Parsippany, NJ 07054<br>+1-973-658-0378	Dana Ilic Bogojevic, MD<br>Safety Physician, Medical Scientific Unit – US<br>Teva Branded Pharmaceutical Products R&D, Inc.<br>400 Interpace Pkwy, Bldg. A, Suite 221<br>Parsippany, NJ 07054<br>+1-973-658-0378	Correction. Updated staff information.
**Appendix A**		
~~Claudio Pasquinelli MD, PhD~~<br>~~Sr Dir Clinical Development, Respiratory~~<br>~~+1-610-893-1264~~<br>Michael J. Lamson, PhD<br>VP, PK and ClinPharm<br>Nuventra, Inc.™<br>2525 Meridian Parkway, Suite 280<br>Durham, North Carolina 27713<br>+1-919-667-7976	Michael J. Lamson, PhD<br>VP, PK and ClinPharm<br>Nuventra, Inc.™<br>2525 Meridian Parkway, Suite 280<br>Durham, North Carolina 27713<br>+1-919-667-7976	Correction. Updated staff information.
~~Haixiao Chen, MD~~<br>~~Global Patient Safety & Pharmacovigilance~~<br>~~+1-610-893-1035~~<br>Dana Ilic Bogojevic, MD<br>Safety Physician, Medical Scientific Unit – US<br>Teva Branded Pharmaceutical Products R&D, Inc.<br>400 Interpace Pkwy, Bldg. A, Suite 221<br>Parsippany, NJ 07054<br>+1-973-658-0378	Dana Ilic Bogojevic, MD<br>Safety Physician, Medical Scientific Unit – US<br>Teva Branded Pharmaceutical Products R&D, Inc.<br>400 Interpace Pkwy, Bldg. A, Suite 221<br>Parsippany, NJ 07054<br>+1-973-658-0378	Correction. Updated staff information.

16.4. Protocol Amendment 01 Dated 02 July 2018

The primary reasons for this amendment are corrections and clarifications. Changes made within the text of the protocol were also made within the synopsis.

Original text with changes shown	New wording	Reason/Justification for change
**Clinical Study Protocol Synopsis**		
**Study :** TV-46046-WH-10147	Study : TV46046-WH-10147	Correction. Dash removed from the study number.
**Section 1.3.2.9 Other**		
Women 40 years or older must have normal mammogram results in the last year prior to enrollment to be eligible for this study. If a woman 40 years or older has not had a mammogram in the past year, one will be scheduled between screening and enrollment.~~Women with normal mammogram results in the last year prior to enrollment will be eligible for this study. If a woman has not had a mammogram in the past year, one will be scheduled between screening and enrollment.~~	Women 40 years or older must have normal mammogram results in the last year prior to enrollment to be eligible for this study. If a woman 40 years or older has not had a mammogram in the past year, one will be scheduled between screening and enrollment.	Clarification. Age requirement added for normal mammograms.
**Section 3.1 General Study Design**		
This is a randomized, crossover, single-center, Phase 1 study to evaluate and compare local tolerability following subcutaneous administration of TV-46046, diluted TV-46046, TV-46046 Placebo, and Depo-subQ 104, in 24 healthy female subjects 18 to 50 years of age~~55 years of age~~.	This is a randomized, crossover, single-center, Phase 1 study to evaluate and compare local tolerability following subcutaneous administration of TV-46046, diluted TV-46046, TV-46046 Placebo, and Depo-subQ 104, in 24 healthy female subjects 18 to 50 years of age.	Correction. Age limit lowered to 50 years of age.
**Section 3.2.1 Subject Inclusion Criteria**		
c. is 18 to ~~55~~ 50 years of age (inclusive)	c. is 18 to 50 years of age (inclusive)	Correction. Age limit lowered to 50 years of age.
f. had a normal mammogram within the last year, if 40 years or older	f. had a normal mammogram within the last year, if 40 years or older	Clarification. Age requirement added for normal mammograms.
**Table 1 Study Procedures and Assessments**		
~~Vital Sign (~~Blood pressure~~)~~ ~~[a]~~	Blood pressure	Correction. Text corrected to remove "Vital signs" and subscript and indicate that blood pressure will be assessed on Day -1.
Vital Signs (Blood pressure, ~~P~~pulse, respiration rate, temperature) [a]	Vital Signs (Blood pressure, pulse, respiration rate, temperature)[a]	Correction. Text corrected to indicate that blood pressure will be assessed on Day 0.

Original text with changes shown	New wording	Reason/Justification for change
[b] Normal mammogram within 1 year prior to enrollment, if 40 years or older	[b] Normal mammogram within 1 year prior to enrollment, if 40 years or older	Clarification. Age requirement added for normal mammograms.
**Section 3.4.1 Procedures for Screening Visit**		
If the participant has not had a mammogram in the past year and is 40 years or older, one ~~should~~ will be scheduled prior to enrollment.	If the participant has not had a mammogram in the past year and is 40 years or older, one will be scheduled prior to enrollment.	Clarification. Age requirement added for normal mammograms.
**Section 3.4.2 Procedures for Enrollment/Injections Visit**		
~~On the day of enrollment, a urine pregnancy test will be performed and weight and vital signs measured (ie, blood pressure, pulse rate, respiration rate and temperature). The enrollment/randomization procedures, including confirming eligibility, should be done after a normal mammogram result has been received.~~<br>On the day of enrollment, a urine pregnancy test will be performed; weight and vital signs will be measured (ie, blood pressure, pulse rate, respiration rate and temperature). Mammogram results from the prior year will be verified for women 40 years and older. Subject eligibility will be confirmed and enrollment/randomization procedures will be performed.	On the day of enrollment, a urine pregnancy test will be performed; weight and vital signs will be measured (ie, blood pressure, pulse rate, respiration rate and temperature). Mammogram results from the prior year will be verified for women 40 years and older. Subject eligibility will be confirmed and enrollment/randomization procedures will be performed.	Clarification. Text edited to improve clarity and include the age requirement for normal mammograms.
**Section 4.6.1 Subject Withdrawal Criteria and Procedures**		
The final visit procedures ~~should~~ will be followed for all subjects who withdraw, if possible (see Section .(3.4.3	The final visit procedures will be followed for all subjects who withdraw, if possible (see Section 3.4.3).	Correction. The word "will" replaced "should".
If the investigator determines that an adverse event is related to the test IMP, monitoring will ~~should~~ continue until the adverse event has resolved or stabilized, the subject exited the study or the subject has reached the end of the follow-up period.	If the investigator determines that an adverse event is related to the test IMP, monitoring will continue until the adverse event has resolved or stabilized, the subject exited the study or the subject has reached the end of the follow-up period.	Correction. The word "will" replaced "should".
**Section 5.1.1 Local Tolerability**		
Photos of the injection site may be taken to supplement documentation of ISRs any time during the study.	Photos of the injection site may be taken to supplement documentation of ISRs any time during the study.	Clarification. Text added to state that photos of the injection site may be taken at any time during the study.

**Original text with changes shown**	**New wording**	**Reason/Justification for change**
All cases of injection site discoloration (hypopigmentation) ~~should~~ will be photographed and consulted by a dermatologist.	All cases of injection site discoloration (hypopigmentation) will be photographed and consulted by a dermatologist.	Correction. The word "will" replaced "should".
**Section 5.2.1 Definition of an Adverse Event**		
In this study, any adverse event occurring after the subject has signed the ICF through the end of the follow up period ~~should~~ will be recorded and reported as an adverse event.	In this study, any adverse event occurring after the subject has signed the ICF through the end of the follow up period will be recorded and reported as an adverse event.	Correction. The word "will" replaced "should".
**Section 5.2.2 Recording and Reporting Adverse Events**		
Serious adverse events occurring in a subject after study discontinuation ~~should~~ will be reported to the sponsor if the investigator becomes aware of them, following the procedures described in Section .5.2.5.3	Serious adverse events occurring in a subject after study discontinuation will be reported to the sponsor if the investigator becomes aware of them, following the procedures described in Section .5.2.5.3	Correction. The word "will" replaced "should".
**5.2.5.3.1 Investigator Responsibility**		
The serious adverse event form ~~should~~ will be sent to FHI 360 (contact information can be found in Appendix A).	The serious adverse event form will be sent to FHI 360 (contact information can be found in Appendix A).	Correction. The word "will" replaced "should".
Serious adverse events occurring to a subject after the treatment of that subject has ended ~~should~~ will be reported to the sponsor if the investigator becomes aware of them.	Serious adverse events occurring to a subject after the treatment of that subject has ended will be reported to the sponsor if the investigator becomes aware of them.	Correction. The word "will" replaced "should".
**Section 5.2.7 Weight and Vital Signs**		
For any abnormal vital sign finding, the measurement ~~should~~ will be repeated	For any abnormal vital sign finding, the measurement will be repeated	Correction. The word "will" replaced "should".
**Section 5.2.8 Medication Error and Special Situations Related to the Investigational Medicinal Products**		
Any administration of IMP that is not in accordance with the study protocol ~~should~~ will be recorded as a deviation.	Any administration of IMP that is not in accordance with the study protocol will be recorded as a deviation.	Correction. The word "will" replaced "should".
If it meets the important protocol deviation criteria, the incorrect IMP administration ~~should~~ will be categorized as "Non-Compliance to Investigational Medicinal Product (IMP)."	If it meets the important protocol deviation criteria, the incorrect IMP administration will be categorized as "Non-Compliance to Investigational Medicinal Product (IMP)."	Correction. The word "will" replaced "should".

Original text with changes shown	New wording	Reason/Justification for change
**Section 11.3 Informed Consent**		
The investigator, or a qualified person designated by the investigator, ~~should~~ will fully inform the subject of all pertinent aspects of the study, including the written information approved by the IRB/IEC. All written and oral information about the study will be provided in a language as nontechnical as practical and understood by the subject. The subject ~~should~~ will be given ample time and opportunity to inquire about details of the study and to decide whether to participate in the study. The above ~~should~~ will be detailed in the source documentation.	The investigator, or a qualified person designated by the investigator, will fully inform the subject of all pertinent aspects of the study, including the written information approved by the IRB/IEC. All written and oral information about the study will be provided in a language as nontechnical as practical and understood by the subject. The subject will be given ample time and opportunity to inquire about details of the study and to decide whether to participate in the study. The above will be detailed in the source documentation.	Correction. The word "will" replaced "should".
**Section 12.1 Source Data and Case Report Forms**		
Subject identity ~~should~~ will not be discernible from the data provided on CRFs.	Subject identity will not be discernible from the data provided on CRFs.	Correction. The word "will" replaced "should".

APPENDIX A. STUDY RESPONSIBILITIES

**Sponsor's Authorized Representative**	Alexandra Kropotova, MD, MBA<br>Vice President, Global Specialty R&D,<br>Respiratory TA Head<br>+1-610-893-1145
**Sponsor's Medical Expert**	Michael J. Lamson, PhD<br>VP, PK and ClinPharm<br>Nuventra, Inc.™<br>2525 Meridian Parkway, Suite 280<br>Durham, North Carolina 27713<br>+1-919-667-7976
**Sponsor's Safety Representative**	Dana Ilic Bogojevic, MD<br>Safety Physician, Medical Scientific Unit – US<br>Teva Branded Pharmaceutical Products R&D, Inc.<br>400 Interpace Pkwy, Bldg. A, Suite 367<br>Parsippany, NJ 07054<br>+1-973-658-0378
**Investigator**	Vivian Brache, Lic<br>Director of the Biomedical Research Department<br>Asociación Dominicana Pro Bienestar de la Familia, Inc.<br>(PROFAMILIA)<br>+1-809-681-8357<br>vbrache@gmail.com
**Coordinating Investigator**	Vera Halpern, MD<br>FHI 360<br>Medical Director<br>+1 919-544-7040 x11390<br>vhalpern@fhi360.org
**Monitor**	FHI 360<br>359 Blackwell Street Ste 200<br>Durham, NC 27701<br>United States
**Trial Supply Management (TSM) Vendor**	CSM<br>342 42nd Street S<br>Fargo ND 58103, USA
**Central Institutional Review Board**	Protection of Human Subjects Committee<br>FHI 360<br>359 Blackwell Street, Suite 200<br>Durham, North Carolina 27701<br>United States of America
